# Single‐subject morphological brain networks: connectivity mapping, topological characterization and test–retest reliability

**DOI:** 10.1002/brb3.448

**Published:** 2016-03-03

**Authors:** Hao Wang, Xiaoqing Jin, Ye Zhang, Jinhui Wang

**Affiliations:** ^1^ Department of PsychologyHangzhou Normal UniversityHangzhou311121China; ^2^ Zhejiang Key Laboratory for Research in Assessment of Cognitive ImpairmentsHangzhou311121China; ^3^ Department of Acupuncture and MoxibustionZhejiang HospitalHangzhou310030China

**Keywords:** Brain network, gray matter volume, hub, reliability, structural MRI

## Abstract

**Introduction:**

Structural MRI has long been used to characterize local morphological features of the human brain. Coordination patterns of the local morphological features among regions, however, are not well understood. Here, we constructed individual‐level morphological brain networks and systematically examined their topological organization and long‐term test–retest reliability under different analytical schemes of spatial smoothing, brain parcellation, and network type.

**Methods:**

This study included 57 healthy participants and all participants completed two MRI scan sessions. Individual morphological brain networks were constructed by estimating interregional similarity in the distribution of regional gray matter volume in terms of the Kullback–Leibler divergence measure. Graph‐based global and nodal network measures were then calculated, followed by the statistical comparison and intra‐class correlation analysis.

**Results:**

The morphological brain networks were highly reproducible between sessions with significantly larger similarities for interhemispheric connections linking bilaterally homotopic regions. Further graph‐based analyses revealed that the morphological brain networks exhibited nonrandom topological organization of small‐worldness, high parallel efficiency and modular architecture regardless of the analytical choices of spatial smoothing, brain parcellation and network type. Moreover, several paralimbic and association regions were consistently revealed to be potential hubs. Nonetheless, the three studied factors particularly spatial smoothing significantly affected quantitative characterization of morphological brain networks. Further examination of long‐term reliability revealed that all the examined network topological properties showed fair to excellent reliability irrespective of the analytical strategies, but performing spatial smoothing significantly improved reliability. Interestingly, nodal centralities were positively correlated with their reliabilities, and nodal degree and efficiency outperformed nodal betweenness with respect to reliability.

**Conclusions:**

Our findings support single‐subject morphological network analysis as a meaningful and reliable method to characterize structural organization of the human brain; this method thus opens a new avenue toward understanding the substrate of intersubject variability in behavior and function and establishing morphological network biomarkers in brain disorders.

## Introduction

The human brain is universally appreciated as a highly integrative system wherein ongoing signaling, information exchange and processing occur in response to a wide variety of endogenous neurophysiological processes and external cognitive demands. There is a growing body of evidence supporting that the brain function is not solely attributable to the dynamics of individual regions but rather integrative processes and dynamic interactions across multiple distributed systems (Bullmore and Sporns 2009; Barch 2013; Sporns 2013, 2014; Cole et al. 2014; Stam 2014; Vertes and Bullmore 2015). This nature makes the brain particularly amenable to study with complex brain network analysis, a powerful tool to investigate how the entire assemblage of brain regions adaptively reorganizes in the face of various cognitive demands and brain disorders (Bullmore and Bassett 2011; Kelly et al. 2012; Borsboom and Cramer 2013; Craddock et al. 2013; Fornito and Bullmore 2015).

Human brain networks (i.e., the connectome) (Sporns et al. 2005; Biswal et al. 2010) can be constructed using multimodal neuroimaging techniques in vivo. Currently, functional MRI (fMRI) and diffusion tensor imaging (DTI) are the two most commonly used approaches to construct individual brain networks by estimating interregional functional connectivity (Biswal et al. 1995; Salvador et al. 2005) or axonal pathways (Hagmann et al. 2007; Iturria‐Medina et al. 2007), respectively. Besides fMRI and DTI, structural MRI (sMRI) has attracted increasing attention recently in delineating whole‐brain morphological connectivity patterns by calculating interregional morphological correlations across a cohort of participants (He et al. 2007; Bassett et al. 2008). Compared with fMRI/DTI, sMRI has distinct advantages in its easy access, high signal‐to‐noise ratio, and relative insensitivity to artifacts (e.g., head motion). Thus, an sMRI‐based network approach is promising to serve as another canonical tool in characterizing network‐level brain organization under both healthy and pathological conditions (Alexander‐Bloch et al. 2013a; Evans 2013). Nevertheless, it should be noted that this methodology can obtain only one network for a group of participants, thereby ignoring interindividual variability and making the examination of brain–behavior relationships and health‐disease classification impossible.

Accordingly, several new methods have been developed to construct individual brain networks based on sMRI data (Raj et al. 2010; Zhou et al. 2011; Tijms et al. 2012). Specifically, in terms of an axon tension theory where axon‐connectivity of cortical areas is believed to have an influence on morphology (Van Essen 1997; Hilgetag and Barbas 2005), Tijms and colleagues proposed an intuitive, test–retest (TRT) reliable method to construct individual morphological networks at a cube (i.e., 3 × 3 × 3 voxels) resolution. Although this cube‐based method keeps the 3D structure of the cortex intact, it ignores remarkable variability of geometry (e.g., shape and size) among different brain regions. Moreover, the rigid extraction of the cubes might not match well with the convolutions of the brain (Tijms et al. 2012). To overcome these limitations, Kong and colleagues introduce a new method to estimate interregional morphological connectivity in terms of the Kullback–Leibler (KL) divergence (Kullback and Leibler 1951) and demonstrate the success of this method in revealing longitudinal changes in morphological connectivity profiles of the thalamus after long‐term sleep deprivation.

**Figure 1:**
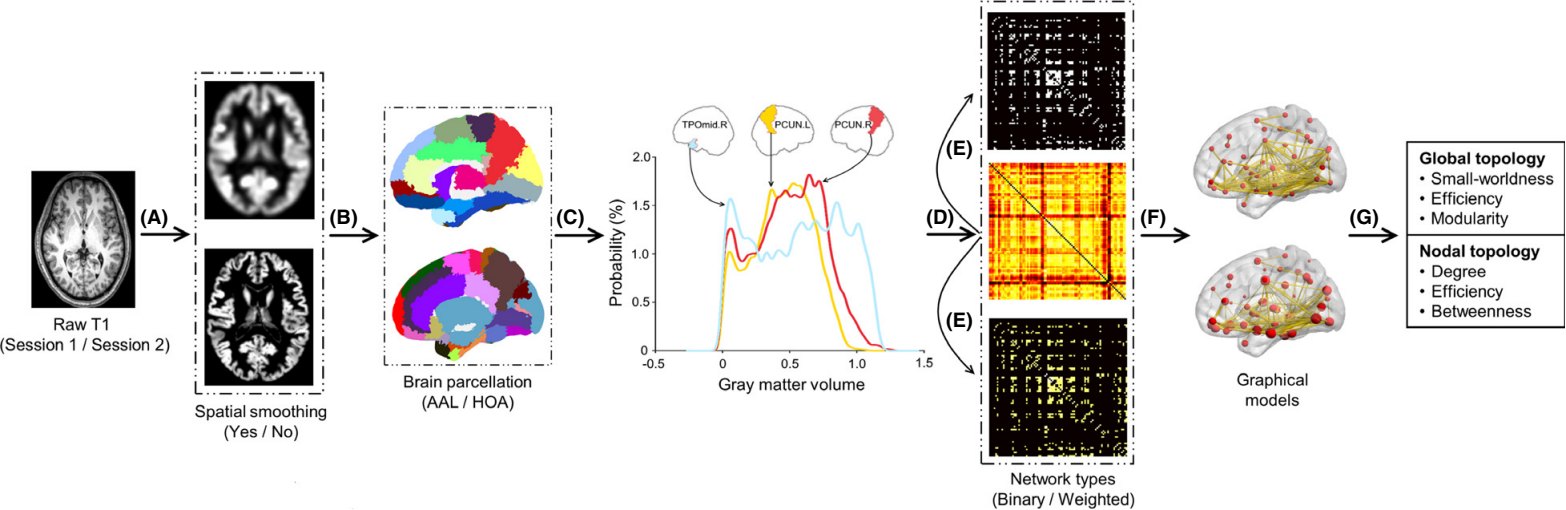
A flowchart illustrating the main analytical process in the current study. Briefly, individual structural images were first segmented into gray matter, white matter and cerebrospinal fluid (A). The gray matter maps (smoothed and nonsmoothed) were then divided into different numbers of regions according to prior brain atlases (AAL and HOA) (B). For each region, the gray matter volume values within it were extracted and used to estimate the probability distribution function (C). Subsequently, the KL divergence‐based similarity was calculated between any pair of regions in their probability distribution functions, resulting in a similarity matrix (D). The resultant similarity matrix was further thresholded into both binary and weighted networks (E), which could be visualized as graphs (F). Finally, several graph‐based network measures were employed to topologically characterize the graphs at both global and nodal levels (G). AAL, Anatomical Automatic Labeling atlas; HOA, Harvard‐Oxford atlas; PCUN, precuneus; TPOmid, temporal pole: middle temporal gyrus; L, left; R, right.

Here, we extended the work of Kong et al. (2014) to construct whole‐brain morphological networks at individual‐level and further characterized their topological organizations at both global and nodal levels. Moreover, we systematically evaluated the influences of several analytical factors on the network topology including spatial smoothing, brain parcellation and network type. Finally, we examined the long‐term TRT reliability of this method in mapping morphological connectivity patterns and capturing their underlying topological architecture. Figure 1 illustrates the schematic representation of the main analytical process used in the current study. Our results showed that the derived morphological brain networks were specifically organized, analytical scheme‐dependent and long‐term TRT reliable, which suggest the current method as a potentially promising framework to associate morphological network organization with interindividual behavior differences in both typical and atypical populations. Notably, a similar study (Kong et al. 2015) was published recently while this work was under peer review. In comparison with what was done by Kong and colleagues, this work has several distinct features, such as the systematic evaluation of effects of different analytical strategies on morphological brain networks (we provide a more detailed comparison of these two studies and results in the discussion).

## Methods

### Participants

A publicly available TRT dataset (1000 Functional Connectomes, RRID: SCR_005361, http://fcon_1000.projects.nitrc.org/indi/CoRR/html/bnu_1.html) was used in the current study, which is a subset of the Connectivity‐based Brain Imaging Research Database at Beijing Normal University. The dataset contained a total of 57 right‐handed participants (male/female: 30/27; age: 19–30 years, mean = 23.09 ± 2.36 years) with no history of neurological and psychiatric disorders. All participants completed two MRI scan sessions (session 1 and session 2) within an interval of approximate 6‐weeks (40.94 ± 4.51 days).

### MRI data acquisition

All MRI scans were performed on a 3T Siemens Tim Trio MRI scanner at the Imaging Center for Brain Research, Beijing Normal University. The T1‐weighted images were acquired using a magnetization prepared rapid gradient echo sequence with the following imaging parameters: repetition time = 2530 ms; echo time = 3.39 ms; inversion time = 1100 ms; slice thickness = 1.33 mm; flip angle = 7°; no interslice gap; 144 sagittal slices covering the whole brain; matrix size = 256 × 256; field of view = 256 × 256 mm^2^. Other modalities were not used in the current study and therefore were not described here.

### Gray matter volume calculation

All data preprocessing (session 1 and session 2) were carried out with the VBM8 toolbox (http://dbm.neuro.uni-jena.de/vbm8) based on Statistical Parametric Mapping 8 (SPM8, RRID: SCR_007037, http://www.fil.ion.ucl.ac.uk/spm/). First, the raw MRI data were checked manually to ensure no obvious artifacts. Then, individual structural MRI images were segmented into gray matter (GM), white matter and cerebrospinal fluid using an adaptive Maximum A Posterior technique. The resultant GM images were subsequently normalized to the MNI space using a high‐dimensional “Diffeomorphic Anatomical Registration Through Exponential Lie Algebra” approach (Ashburner 2007) and further nonlinearly modulated to compensate for spatial normalization effects. The nonlinear modulation essentially corrected for individual differences in brain size. After these steps, a GM volume map was obtained for each participant (1.5 mm isotropic voxels).

### Spatial smoothing

Spatial smoothing is a typically used step for voxel‐based morphology analysis that increases the signal‐to‐noise ratio and improves intersubject anatomical correspondence of sulci and gyri in the brain. However, on the other hand, this step may introduce spurious local anatomical connectivity (see below for the definition of anatomical connectivity in the current study) due to the fusion of signals for spatially adjacent regions. Considering that little is known about the effects of spatial smoothing on the subsequent connectivity mapping and topological characterization, we performed all the following analyses separately for GM volume maps with or without spatial smoothing (Gaussian kernel with 6‐mm full width at half maximum).

### Construction of individual morphological brain networks

In the current study, we constructed large‐scale morphological brain networks for each participant based on their GM volume images. A brain network is comprised of a collection of nodes and edges interconnecting the nodes, wherein nodes represent brain regions and edges represent interregional similarity in the distributions of regional GM volume here.

#### Definition of network nodes

To define network nodes or brain regions, we parcellated the brain into different regions of interest (ROIs) in terms of prior brain atlases. A convergent finding from different neuroimaging modalities has shown that different brain parcellation schemes are associated with different topological organizations for the resultant brain networks (Wang et al. 2009; Sanabria‐Diaz et al. 2010; Zalesky et al. 2010). Thus, we employed two widely used structural brain templates of the Anatomical Automatic Labeling atlas (AAL, RRID: SCR_003550) (Tzourio‐Mazoyer et al. 2002) and the Harvard‐Oxford atlas (HOA, RRID: SCR_001476) (Kennedy et al. 1998; Makris et al. 1999) to divide the brain into different numbers of ROIs (90 for the AAL in Table S1 and 112 for the HOA in Table S2). This allows us to estimate the robustness of our findings against different parcellation schemes.

#### Definition of network edges

To estimate internodal or interregional network edges, we utilized a KL divergence‐based similarity (KLS) measure to quantify morphological connectivity between two regions (Kong et al. 2014). For each participant, we first extracted GM volume values for all the voxels within each ROI under each brain parcellation scheme. The probability density function of these values was then estimated using the kernel density estimation (KDE) (Rosenblatt 1956; Parzen 1962) with bandwidths chosen automatically (Botev et al. 2010). This analysis was performed using public Matlab code posed by Botev (function: kde, http://www.mathworks.com/matlabcentral/fileexchange/14034-kernel-density-estimator). Further, the probability distribution function (PDF) was calculated for the obtained probability density function. Subsequently, the KL divergence was calculated between any pair of ROIs in their PDFs. KL divergence is an index from probability theory to measure the difference between two probability distributions or the information lost when a probability distribution is used to approximate another from the perspective of information theory (Burnham and Anderson 2002). Formally, the KL divergence from distribution *Q* to *P* is defined as:

$$D_{\mathrm{KL}}\left(P||Q\right)=\sum_{i=1}^{n}P\left(i\right)\log\frac{P(i)}{Q(i)},$$

where *P* and *Q* are two PDFs, *n* is the number of sample points (see “Results” for the selection of *n* in the current study). It is worth noting that $D_{\mathrm{KL}}\left(P||Q\right)$ is not equal to $D_{\mathrm{KL}}\left(Q||P\right)$. To derive a symmetric measure, we calculated a variation of the KL divergence as follows:

$$D_{\mathrm{KL}}\left(P,Q\right)=\sum_{i=1}^{n}\left(P\left(i\right)\log\frac{P(i)}{Q(i)}+Q\left(i\right)\log\frac{Q(i)}{P(i)}\right).$$

Finally, the KLS is computed as:

$$\text{KLS}\;\left(P,Q\right)=e^{-D_{\mathrm{KL}}\left(P,Q\right)},$$

where *e* is nature exponential. Through this transformation, the KLS ranges from 0 to 1, where 1 is for two identical distributions. After all these analyses, four KLS‐based morphological connectivity matrices were generated for each participant (self‐connections were set to 0): one for AAL‐based parcellation on smoothed GM maps, one for AAL‐based parcellation on unsmoothed GM maps, one for HOA‐based parcellation on smoothed GM maps and the other for HOA‐based parcellation on unsmoothed GM maps.

#### Determination of the number of sampling points during KDE

Before application of KDE to human brain data, it is important to investigate how many sampling points are needed to generate stable estimation. Therefore, we first examined the effects of different numbers of sampling points on the estimation of regional probability density function. Specifically, we estimated the probability density function for each region of each participant under each combination between spatial smoothing and brain parcellation atlas when different numbers of sampling points were used (2^4^, 2^5^, 2^6^, 2^7^, 2^8^, 2^9^ and 2^10^). Then, the Fréchet distance (Alt and Godau 1995) was calculated for each region between any pair of probability density functions that were estimated using adjacent numbers of sampling points (i.e., 2^4^ and 2^5^, 2^5^ and 2^6^, 2^6^ and 2^7^, 2^7^ and 2^8^, 2^8^ and 2^9^, and 2^9^ and 2^10^). The Fréchet distance provides a measure to quantify the similarity/dissimilarity between two curves that not only takes into account the location and ordering of the points along the curves but also can deal with curves with different lengths. After this procedure, two 57 (participants) × 90 (regions) × 6 (adjacent pairs in sampling number) Fréchet distance matrices were obtained when the AAL‐based parcellation was used: one for smoothed data, the other for nonsmoothed data. Similarly, two 57 (participants) × 112 (regions) × 6 (adjacent pairs in sampling number) Fréchet distance matrices were obtained when the HOA‐based parcellation was used. Subsequently, the four matrices were separately averaged along the first dimension (i.e., participants) to derive four group‐level, regional mean Fréchet distance matrices (two 90 × 6, the other two 112 × 6). Finally, any pair of nearby columns of each the resultant matrix was compared by using a nonparametric permutation test (10,000 iterations). We found that the differences in the regional mean Fréchet distances between nearby columns became nonsignificant from the comparison between column 3 and column 4 for all the combinations between spatial smoothing and brain parcellation (Table 1). This means that the similarities/dissimilarities between regional probability density functions estimated with 2^6^ and 2^7^ sampling points (i.e., column 3) were comparable with those derived from 2^7^ and 2^8^ sampling points (i.e., column 4), and so on. Accordingly, 2^7^ sampling points was conservatively chosen in the current study to provide a trade‐off between stable estimation of regional probability density functions and computational complexity.

**Table 1:** The *P* values of pairwise comparisons of the Fréchet distances between curves estimated with nearby sample points

Categories	A versus B	B versus C	C versus D	D versus E	E versus F
Nonsmo‐AAL	0.016	0.051	0.215	0.451	0.071
Nonsmo‐HOA	0.477	0.432	0.439	0.390	0.272
Smo‐AAL	0.011	0.133	0.135	0.155	0.174
Smo‐HOA	0.005	0.012	0.093	0.135	0.211

Nonsmo, no spatial smoothing; Smo, spatial smoothing; AAL, Anatomical Automatic Labeling atlas; HOA, Harvard‐Oxford atlas. A – the Fréchet distances between curves estimated with 2^4^ and 2^5^ sample points; B – the Fréchet distances between curves estimated with 2^5^ and 2^6^ sample points; C – the Fréchet distances between curves estimated with 2^6^ and 2^7^ sample points; D – the Fréchet distances between curves estimated with 2^7^ and 2^8^ sample points; E – the Fréchet distances between curves estimated with 2^8^ and 2^9^ sample points; F – the Fréchet distances between curves estimated with 2^9^ and 2^10^ sample points.

#### Threshold selection

Prior to topological characterization of the derived morphological connectivity matrices, a thresholding procedure is typically used to exclude noisy elements. Here, we employed a sparsity threshold, *S* (defined as the ratio of the number of actual edges divided by the maximum possible number of edges in a network) to convert each matrix *C*_*ij*_ = [*c*_*ij*_] into a binary and a weighted network by applying a subject‐specific KLS threshold:

$$A_{ij}=\left[a_{ij}\right]=\left\{\begin{array}{c} 1,\;\text{if}\;c_{ij}>{\text{KLS}}_{\mathrm{thr}};\\ 0,\;\text{others} \end{array}\right.$$

and a weighted network

$$W_{ij}=\left[w_{ij}\right]=\left\{\begin{array}{c} c_{ij},\;\text{if}\;c_{ij}>{\text{KLS}}_{\mathrm{thr}};\\ 0,\;\text{others} \end{array}\right.$$

This thresholding method ensures the same number of nodes and edges for the resultant networks across participants. Due to the lack of a conclusive method to select a single threshold, a consecutive sparsity range of 0.05 < *S *<* *0.4 (interval = 0.02) was chosen, where the resultant networks have sparse properties (Achard et al. 2006; He et al. 2007; Wang et al. 2009) and are estimable for the small‐world attributes (Watts and Strogatz 1998). All the following network analyses were performed at each of the threshold level in this range, therefore resulting in functions or curves of sparsity for the topological measures listed below.

### Network analysis

Based on the analyses above, we obtained 8 = 2 (spatial smoothing: yes vs. no) × 2 (brain parcellation: AAL vs. HOA) × 2 (network type: binary vs. weighted) morphological brain networks at each sparsity level for each participant. For each of these networks, we calculated both global (clustering coefficient, *C*_p_, characteristic path length, *L*_p_, local efficiency, *E*_loc_, global efficiency, *E*_glob_ and modularity, *Q*) and nodal (nodal degree, *k*_i_, nodal efficiency, *e*_i_ and nodal betweenness, *b*_i_) metrics with the GRETNA toolbox (Wang et al. 2015a). Detailed formulas, usages and explanations of these metrics in the brain networks can be found in our previous study (Wang et al. 2011) and in an excellent methodological review (Rubinov and Sporns 2010).

To determine whether the morphological brain networks were nonrandomly organized, all the global network measures were separately normalized by the corresponding mean of 100 matched random networks. The random networks were generated using a topological rewiring algorithm (Maslov and Sneppen 2002) which preserved the same number of nodes and edges and the same degree distribution as real brain networks. Typically, an efficient, small‐world and modular network should fulfill the following conditions: normalized *E*_loc_ > 1 and normalized *E*_glob_ ~ 1, normalized *C*_p_ > 1 and normalized *L*_p_ ~ 1 and normalized *Q* > 1. Notably, to simplify subsequent TRT reliability and statistical analyses, we also calculated the area under curve (AUC, i.e., the integral over sparsity range) to provide a threshold‐independent summary scalar for each global and nodal network metric of each participant under each analytical combination

### TRT reliability

We utilized a common index of intra‐class correlation (ICC) (Shrout and Fleiss 1979) to investigate the TRT reliability of the current single‐subject method in mapping morphological connectivity patterns and characterizing their topological organization. Specifically, for each connectivity element (i.e., KLS value) or network metric (global or nodal) derived from each appropriate combination of the three factors mentioned above, individual values were first merged into a 57 × 2 matrix with rows corresponding to participants and columns corresponding to sessions. Using a one‐way analysis of variance (ANOVA), we then split the total sum of the squares into between‐subject (MS_b_) and within‐subject (MS_w_, i.e., residual error) sum of squares. The ICC was then calculated as:

$$\text{ICC}=\frac{{\text{MS}}_{b}-{\text{MS}}_{w}}{{\text{MS}}_{b}+\left(k-1\right)\;{\text{MS}}_{w}},$$

where *k* is the number of repeated observations per participant (2 here). ICC is close to 1 for reliable measures that show low within‐subject variance relative to between‐subject variance and 0 (negative) otherwise. Consistent with our previous study (Wang et al. 2011), the reliability was categorized into poor (0 < ICC < 0.25), low (0.25 < ICC < 0.4), fair (0.4 < ICC < 0.6), good (0.6 < ICC < 0.75) and excellent (0.75 < ICC < 1.0).

### Statistical analysis

To determine whether different analytical factors of spatial smoothing, brain parcellation and network type significantly affect topological descriptions of the morphological networks, a three‐way repeated measures ANOVA was performed for each global network parameter (clustering coefficient, characteristic path length, local efficiency, global efficiency and modularity and their corresponding normalization versions). For nodal centralities (nodal degree, efficiency and betweenness), a two‐way repeated measures ANOVA was performed for each nodal metric under the AAL and HOA schemes, respectively, due to the different numbers of nodes. Analogously, the three‐way and two‐way repeated measures ANOVA were separately conducted to examine the effects of different analytical strategies on the TRT reliability (ICC values) of global and nodal network measures.

## Results

### KLS‐based morphological brain networks

#### Patterns of morphological similarity matrices

**Figure 2:**
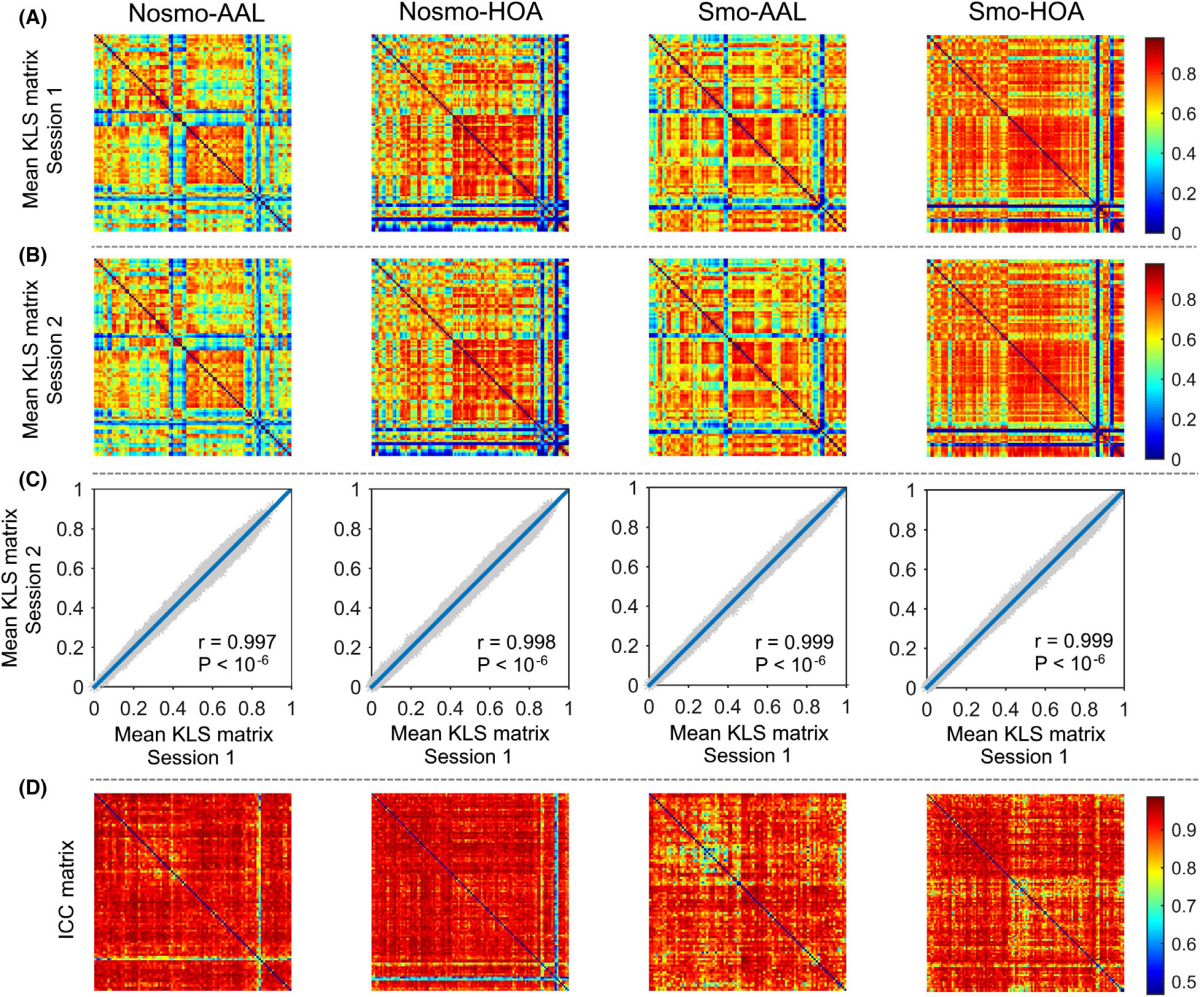
Spatial pattern, intersession similarity and TRT reliability of KLS‐based morphological connectivity matrices. The mean KLS matrices (across participants) derived from session 1 (A) and session 2 (B) were highly correlated with each other, a robust finding against different choices of spatial smoothing and brain parcellation (C). TRT reliability analysis revealed high reliability for most elements in the matrices (D). KLS, KL divergence‐based similarity; ICC, intra‐class correlation; Nosmo, no smoothing; Smo, smoothing; AAL, Anatomical Automatic Labeling atlas; HOA, Harvard‐Oxford atlas.

Figure 2A and B show the mean morphological similarity matrices under all combinations between spatial smoothing and brain parcellation scheme for data session 1 and session 2, respectively. Visual inspection found that the connectivity patterns were complex but specifically organized with strong interhemispheric morphological similarity between bilaterally homologous regions. This was further validated by statistical analyses showing that the similarity values between homotopic regions were significantly greater than the others in the matrix, a robust finding against the analytical choices of spatial smoothing, brain parcellation scheme and data session (*t*‐test, all *P* < 10^−3^). Moreover, quantitative spatial correlation analyses showed that the overall patterns of morphological similarity matrices were highly similar between session 1 and session 2 at both group (*r* > 0.99) (Fig. 2C) and individual levels (mean *r* > 0.91) regardless of the analytical strategies used.

#### TRT reliability of morphological similarity matrices

Intra‐class correlation‐based TRT reliability analysis on individual elements in the morphological similarity matrices demonstrated high reliability for interregional KLS values (Nosmo‐AAL: 0.816 ± 0.098; Nosmo‐HOA: 0.816 ± 0.140; Smo‐AAL: 0.905 ± 0.050; Smo‐HOA: 0.899 ± 0.056) (Fig. 2D). Specifically, under each brain parcellation scheme, more than 85% elements derived from nonsmoothed data and more than 97% elements derived from smoothed data exhibited excellent reliability. However, in contrast to the abovementioned finding of higher similarities for interhemispheric connections between bilaterally homologous regions, lower TRT reliability were found for these connections than the others in the matrices regardless of the analytical choices of spatial smoothing and brain parcellation scheme (*t*‐test, all *P* < 10^−3^). This implies that the similarities between bilaterally homologous regions are related to higher within‐subject or lower between‐subject variance.

### Global organization of morphological brain networks

#### Small‐worldness, efficiency and modularity

**Figure 3:**
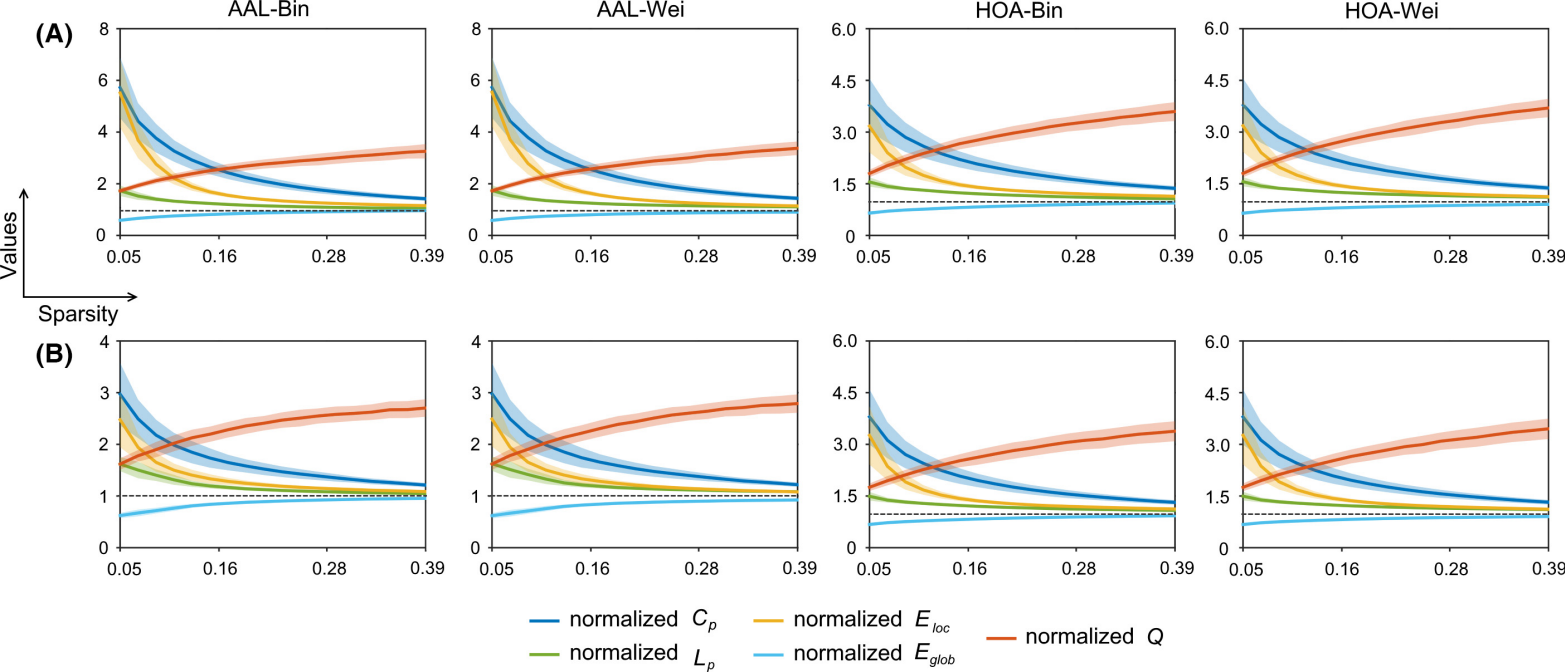
The normalized global network measures as a function of sparsity for morphological brain networks from nonsmoothed (A) and smoothed (B) data. All the normalized global network measures significantly deviated from 1 (dotted lines) regardless of different analytical strategies, indicating obviously different organizations of morphological brain networks from matched random networks. The results were from data session 1. Bin, binary network; Wei, weighted network; AAL, Anatomical Automatic Labeling atlas; HOA, Harvard‐Oxford atlas.

**Figure 4:**
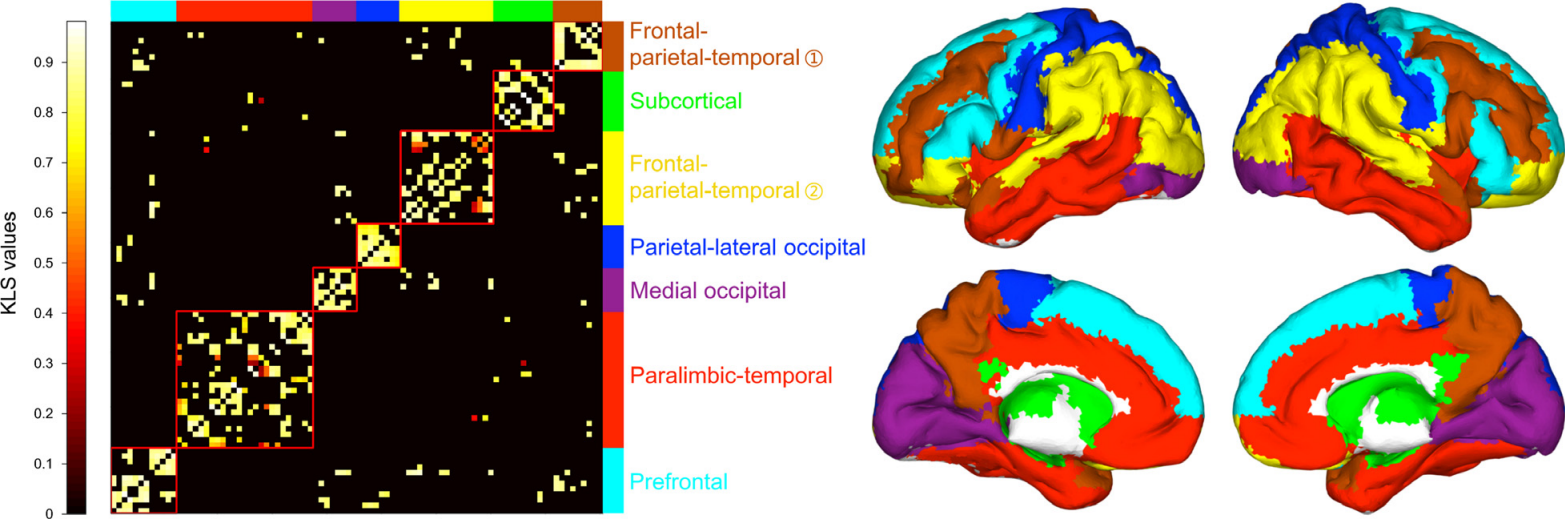
Matrix and brain surface presentations of modular structure for morphological brain networks. A group‐level backbone matrix (left) was derived from the mean morphological brain network across all participants based on spatially smoothed data under the AAL parcellation scheme. The backbone matrix was further reordered according to the optimal modular partition as shown in the brain surface (right). Seven modules were identified that corresponded well with known neuroanatomical systems, such as the medial occipital module and subcortical module. KLS, Kullback–Leibler divergence‐based similarity. AAL, Anatomical Automatic Labeling atlas.

Compared with random networks, the morphological brain networks exhibited larger values in the clustering coefficient, local efficiency and modularity but approximately equal values in the characteristic path length and global efficiency under each analytical combination of spatial smoothing, network type and brain parcellation. This resulted in a pattern of normalized clustering coefficient, local efficiency and modularity > 1 and normalized characteristic path length and global efficiency ~ 1 (Fig. 3). These findings suggest conserved global network organization of high‐efficient, small‐world and modular architectures for morphological brain networks. In addition, we also presented the modular structure derived from the group‐level mean morphological brain network based on spatially smoothed data under the AAL parcellation scheme (Fig. 4). Seven modules were found (*Q *=* *0.628, *z*‐score = 27.030) that corresponded well with known neuroanatomical systems, and the modules were similar to those derived from diffusion and functional MRI data, such as the visual module of (medial) occipital system and subcortical module (Chen et al. 2008; Hagmann et al. 2008; He et al. 2009b; Meunier et al. 2009).

#### Effects of spatial smoothing, brain parcellation and network type on global network measures

**Figure 5:**
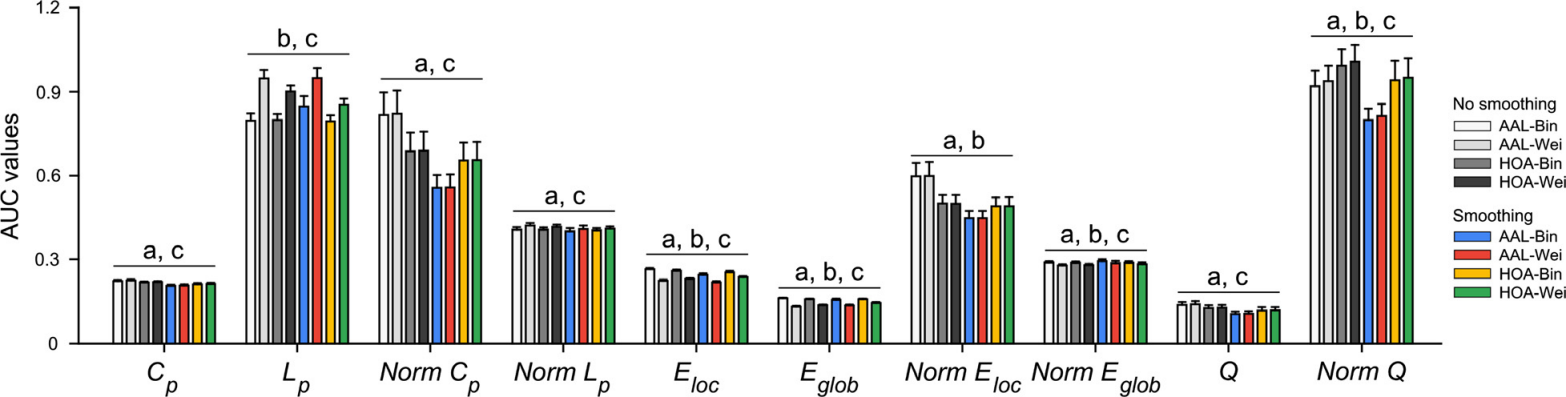
Effects of spatial smoothing, brain parcellation and network type on global network measures. The statistical analysis was done for each global network measure based on their areas under curve. Generally, the three factors significantly influenced quantitative descriptions of all the studied global network measures in a complex pattern. These results were from data session 1. The results were from data session 1. AUC, the area under curve; Bin, binary network; Wei, weighted network; AAL, Anatomical Automatic Labeling atlas; HOA, Harvard‐Oxford atlas. a, significant main effect of spatial smoothing; b, significant main effect of brain parcellation; c, significant main effect of network type. Of note, all the interactive effects were also significant.

Generally, the results showed that the three factors significantly influenced numerical values of all the global network measures in a complex interactive pattern (Fig. 5). This suggests that quantitative characterization of morphological brain networks depends on the analytical strategies. No further post hoc analyses were done because of the complex patterns and more importantly comparisons of numerical values make no sense regarding the selection of optimal analytical strategy.

#### TRT reliability of global network measures

**Figure 6:**
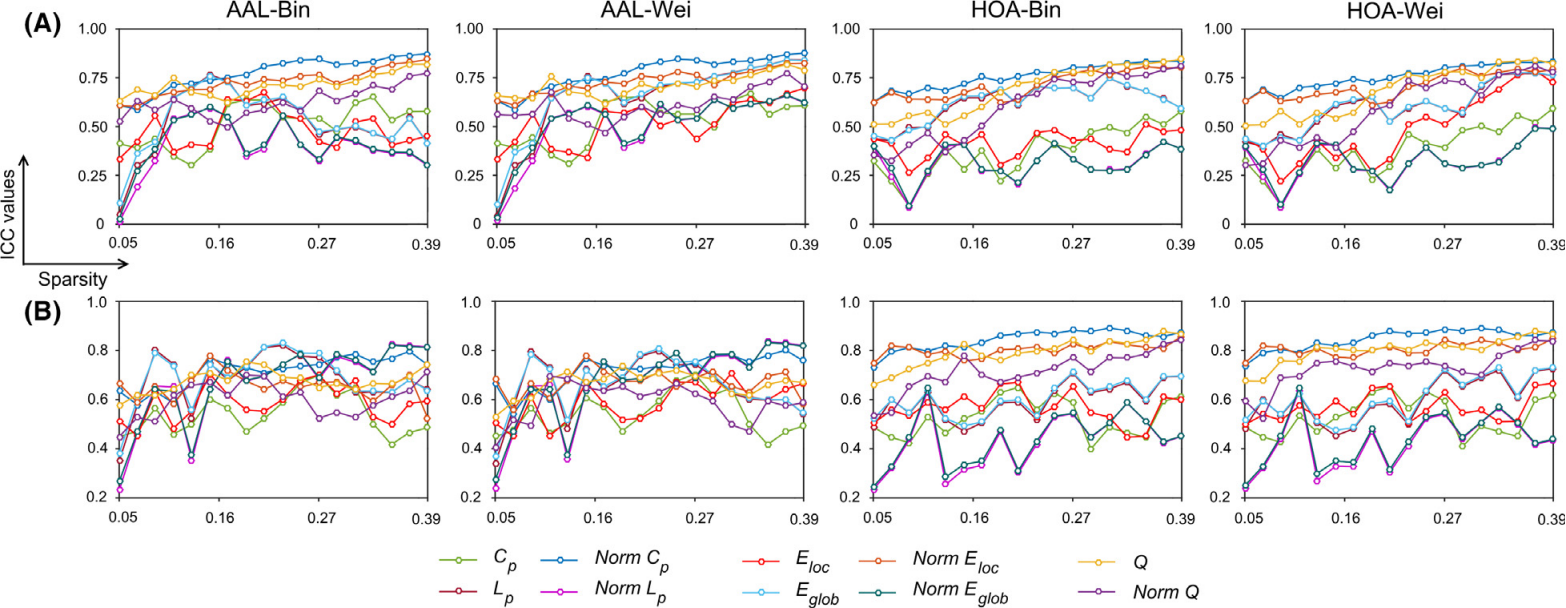
The test–retest (TRT) reliability of global network measures as a function of sparsity for morphological brain networks from nonsmoothed (A) and smoothed (B) data. Generally, despite of different analytical strategies, the global network measures showed fair to excellent reliabilities (i.e., 0.4 < ICC < 1) over almost the whole sparsity range studied. Bin, binary network; Wei, weighted network; AAL, Anatomical Automatic Labeling atlas; HOA, Harvard‐Oxford atlas; ICC, intra‐class correlation.

**Figure 7:**
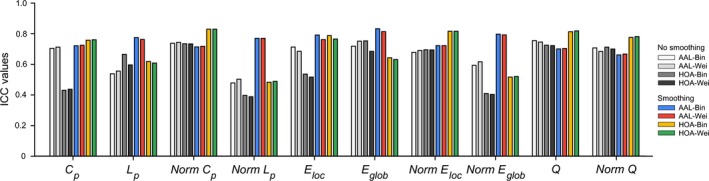
The test–retest (TRT) reliability of global network measures based on the area under curve. Fair to excellent reliabilities (i.e., 0.4 < ICC < 1) were found for all global network measures irrespective of different choices of spatial smoothing, brain parcellation and network type. ICC, intra‐class correlation; Bin, binary network; Wei, weighted network; AAL, Anatomical Automatic Labeling atlas; HOA, Harvard‐Oxford atlas.

Figure 6 shows the TRT reliability for all the global network measures as a function of sparsity threshold. All the global measures exhibited fair to good reliability (i.e., ICC ranged from 0.40 to 0.75) over most of the sparsity range studied (mean ICC over sparsity threshold and across network measures ranged from 0.523 to 0.648). Further analysis based on the AUCs revealed even higher reliability for the global network measures (mean ICC across network measures ranged from 0.613 to 0.781) (Fig. 7). In addition, we compared the reliability between original or first‐order global network measures (clustering coefficient, characteristic path length, local efficiency, global efficiency and modularity) and their normalized or second‐order versions and found no significant difference (*P *>* *0.05).

#### Effects of spatial smoothing, brain parcellation and network type on TRT reliability of global network topology

To examine whether the ICC of global network topology depends on different analytical strategies, a three‐way repeated ANOVA measures was conducted across different network measures. The results showed that only spatial smoothing significantly affected the reliability of global network measures (*F*_1,9_ = 17.543, *P *=* *0.002). No effects were observed for brain parcellation, network type or any interaction (*P *>* *0.05). Post hoc comparisons showed that performing spatial smoothing significantly improved the reliability of global network measures (*t*_78_ = 3.848, *P* < 10^−3^).

### Local nodal characteristics of morphological brain networks

#### Hubs

**Figure 8:**
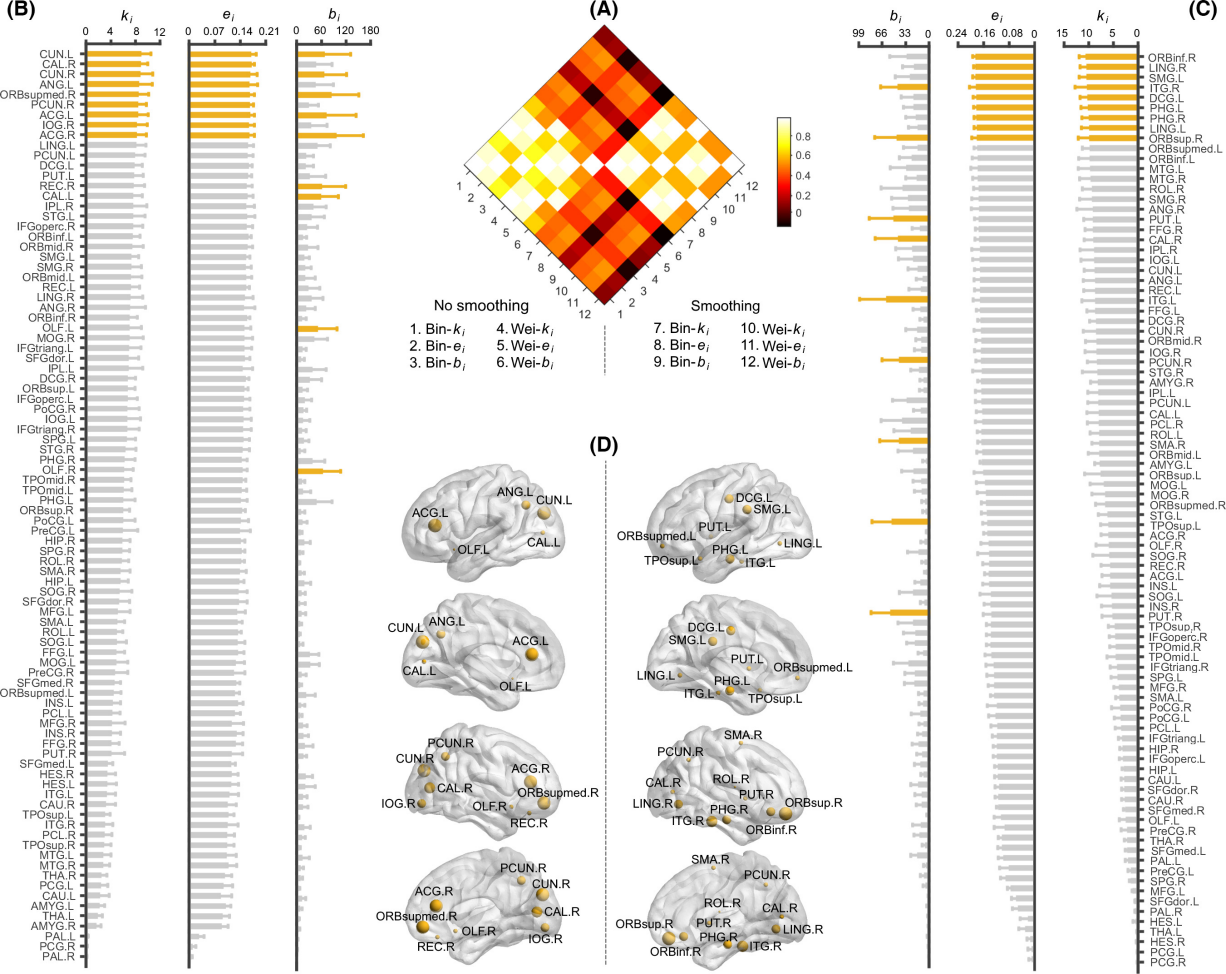
Nodal centralities under the AAL parcellation scheme. Pairwise correlation analyses revealed that spatial smoothing significantly modulated spatial patterns of nodal centralities (A). Therefore, (B) and (C) were used to illustrate specific patterns of nodal centralities (weighted network analysis) for nonsmoothed and smoothed data, respectively. Regions with the highest centralities (top 10%) were highlighted in orange. A hub score was further calculated to identify regions that consistently showed high centralities over different network types and centrality metrics (D; left for no n‐smoothed data and right for smoothed data; also see Table 2). The results were from data session 1. All regional abbreviations can be found in Table S1. AAL, Anatomical Automatic Labeling atlas.

**Figure 9:**
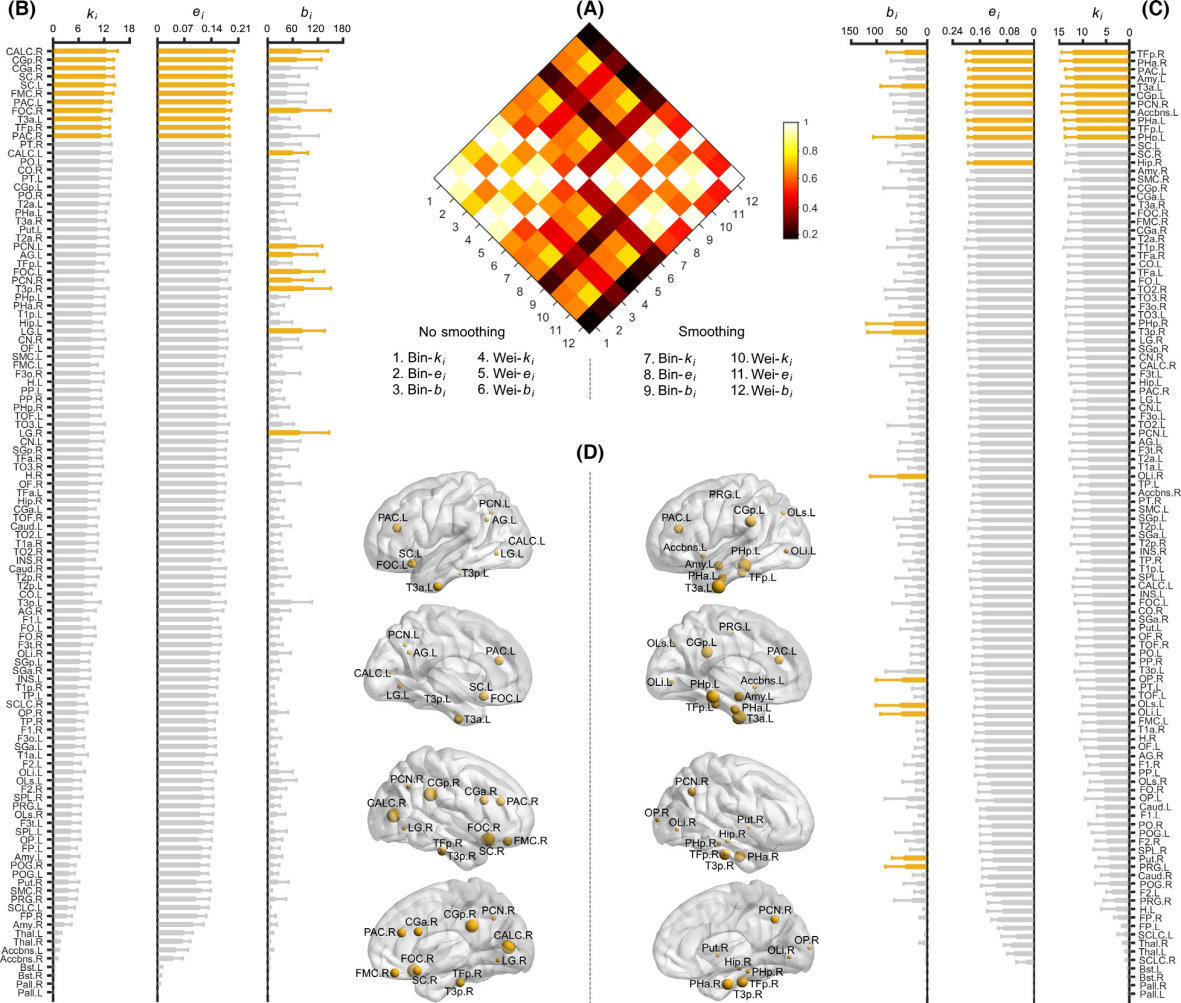
Nodal centralities under the HOA parcellation scheme. The results were presented in a similar manner to that in Fig. 8. All regional abbreviations can be found in Table S2. HOA, Harvard‐Oxford atlas.

All nodal results were based on the AUCs. Therefore, a total of 12 = 2 (spatial smoothing: yes and no) × 2 (network type: binary and weighted) × 3 (nodal centrality metrics: degree, efficiency and betweenness) nodal centrality maps were obtained for each participant under each brain parcellation scheme (AAL and HOA). We first calculated the Pearson correlations between any pair of these nodal centrality maps after averaging them across participants. This resulted in a 12 × 12 correlation matrix under the AAL and HOA parcellation scheme, respectively (Figs. 8A, 9A). We found that the correlation coefficients were significantly larger for nonsmoothed (AAL: 0.786 ± 0.179; HOA: 0.791 ± 0.170) and smoothed (AAL: 0.742 ± 0.213; HOA: 0.748 ± 0.208) data than those between nonsmoothed and smoothed data (AAL: 0.299 ± 0.206; HOA: 0.473 ± 0.188) (permutation test, 10,000 iterations, *P *<* *0.001 for all comparisons). These findings indicate that spatial smoothing significantly affects the distribution of nodal centralities in the brain for morphological brain networks. Therefore, we separately presented nodal centralities for nonsmoothed (Fig. 8B for AAL and Fig. 9B for HOA) and smoothed (Fig. 8C for AAL and Fig. 9C for HOA) data and highlighted the top 10% regions (9 for AAL and 11 for HOA) with the highest centrality values, which may serve as potential hub regions.

To further locate brain regions that were consistently identified as hubs, we assigned each node a hub‐score (ranged from 0 to 6) indicating the times that the node fell within the top 10% nodes across nodal centrality metrics and network types. For example, if a region was identified as a hub by all the nodal centrality metrics (degree, efficiency and betweenness) of both binary and weighted networks, its hub‐score was 6. Figures 8D, 9D show the spatial distributions of nodal hub‐scores in the brain under the brain parcellation schemes of AAL and HOA, respectively. The most consistent hubs (scored > 3), as summarized in Table 2, were mainly limbic/paralimbic and association cortices but with a slight overlap between nonsmoothed and smoothed data irrespective of the brain parcellation schemes.

**Table 2:** Regions consistently showing high centralities (i.e., hubs)

Name	Score	Category	Name	Score	Category
Nonsmo‐AAL			Smo‐AAL		
Superior frontal gyrus, medial orbital (R)	6	Paralimbic	Superior frontal gyrus, orbital part (R)	6	Paralimbic
Anterior cingulate and paracingulate gyri (L)	6	Paralimbic	Inferior temporal gyrus (R)	5	Association
Anterior cingulate and paracingulate gyri (R)	6	Paralimbic	Inferior frontal gyrus, orbital part (R)	4	Paralimbic
Cuneus (L)	6	Association	Median cingulate and paracingulate gyri (L)	4	Paralimbic
Cuneus (R)	6	Association	Parahippocampal gyrus (L)	4	Paralimbic
Calcarine fissure and surrounding cortex (R)	5	Primary	Parahippocampal gyrus (R)	4	Paralimbic
Inferior occipital gyrus (R)	4	Association	Lingual gyrus (R)	4	Association
Angular gyrus (L)	4	Association	Supramarginal gyrus (L)	4	Association
Precuneus (R)	4	Association			
Nonsmo‐HOA			Smo‐HOA		
Cingulate gyrus, posterior division (R)	6	Paralimbic	Inferior temporal gyrus, anterior division (L)	6	Association
Frontal orbital cortex (R)	6	Paralimbic	Parahippocampal gyrus, posterior division (L)	6	Paralimbic
Intracalcarine cortex (R)	6	Primary	Cingulate gyrus, posterior division (R)	5	Paralimbic
Inferior temporal gyrus, anterior division (L)	4	Association	Parahippocampal gyrus, anterior division (R)	5	Paralimbic
Temporal fusiform cortex, posterior division (R)	4	Association	Temporal fusiform cortex, posterior division (R)	5	Association
Frontal medial cortex (R)	4	Paralimbic	Temporal fusiform cortex, posterior division (L)	4	Association
Subcallosal cortex (L)	4	Limbic	Amygdala (L)	4	Subcortical
Subcallosal cortex (R)	4	Limbic	Paracingulate gyrus (L)	4	Paralimbic
Paracingulate gyrus (L)	4	Paralimbic	Precuneous cortex (R)	4	Association
Paracingulate gyrus (R)	4	Paralimbic	Parahippocampal gyrus, anterior division (L)	4	Paralimbic
Cingulate gyrus, anterior division (R)	4	Paralimbic			

Nonsmo, no smoothing; Smo, smoothing; AAL, Anatomical Automatic Labeling atlas; HOA, Harvard‐Oxford atlas; L, left; R, right.

#### Effects of spatial smoothing and network type on nodal centralities

A two‐way repeated ANOVA was separately performed to test the effects of spatial smoothing and network type on the mean (across participants) values of nodal degree, efficiency and betweenness. The results showed that no matter which parcellation scheme was used, network type significantly affected nodal degree and efficiency (*P *<* *0.05, corrected by false discovery rate procedure across centrality metrics) but not nodal betweenness, and the effects were further modulated by the other factor of spatial smoothing. Again, no further post hoc analyses were needed here.

#### TRT reliability of nodal centralities

**Figure 10:**
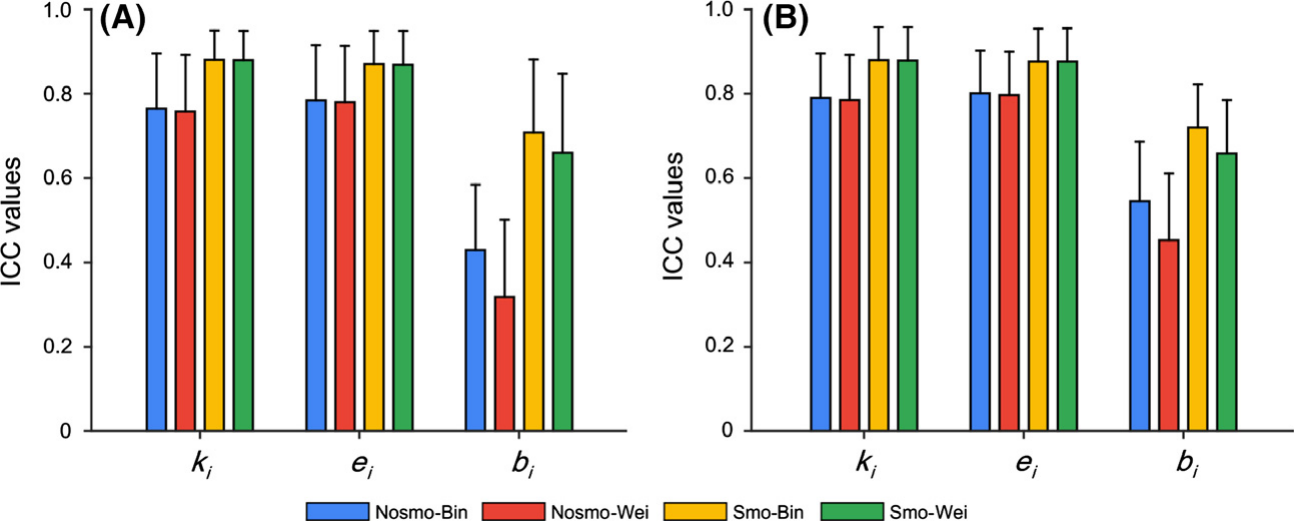
The test–retest (TRT) reliability of nodal centrality metrics under the Anatomical Automatic Labeling atlas (AAL) (A) and Harvard‐Oxford atlas (HOA) (B) parcellation schemes. Fair to excellent reliability (i.e., 0.4 < ICC < 1) was observed for almost all nodal centrality metrics under all analytical combinations of spatial smoothing, brain parcellation and network type. Notably**,** nodal degree and efficiency exhibited obviously higher reliability than nodal betwenness regardless of the parcellation schemes. ICC, intra‐class correlation; Bin, binary network; Wei, weighted network; Nosmo, no smoothing; Smo, smoothing.

**Figure 11:**
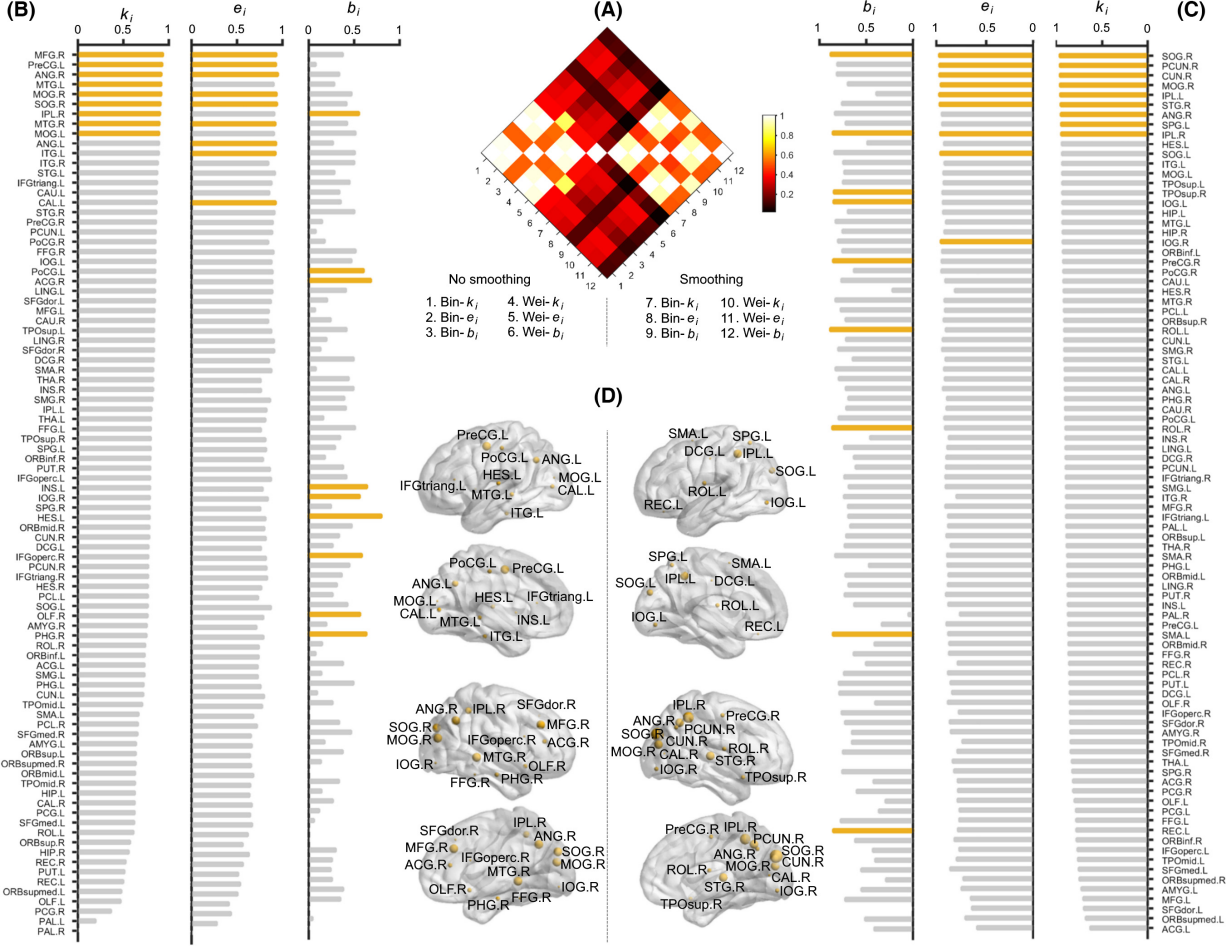
The test–retest (TRT) reliability of nodal centralities under the Anatomical Automatic Labeling atlas (AAL) parcellation scheme. Pairwise correlation analyses revealed that spatial smoothing significantly modulated spatial patterns of nodal reliabilities (A). Therefore, (B) and (C) were used to illustrate specific patterns of nodal reliabilities (weighted network analysis) for nonsmoothed and smoothed data, respectively. Regions with the highest reliabilities (top 10%) were highlighted in orange. A reliability‐based hub score was further calculated to identify regions that consistently showed high reliability over different network types and centrality metrics (D; left for nonsmoothed data and right for smoothed data; also see Table 3). The results were from data session 1. All regional abbreviations can be found in Table S1.

**Figure 12:**
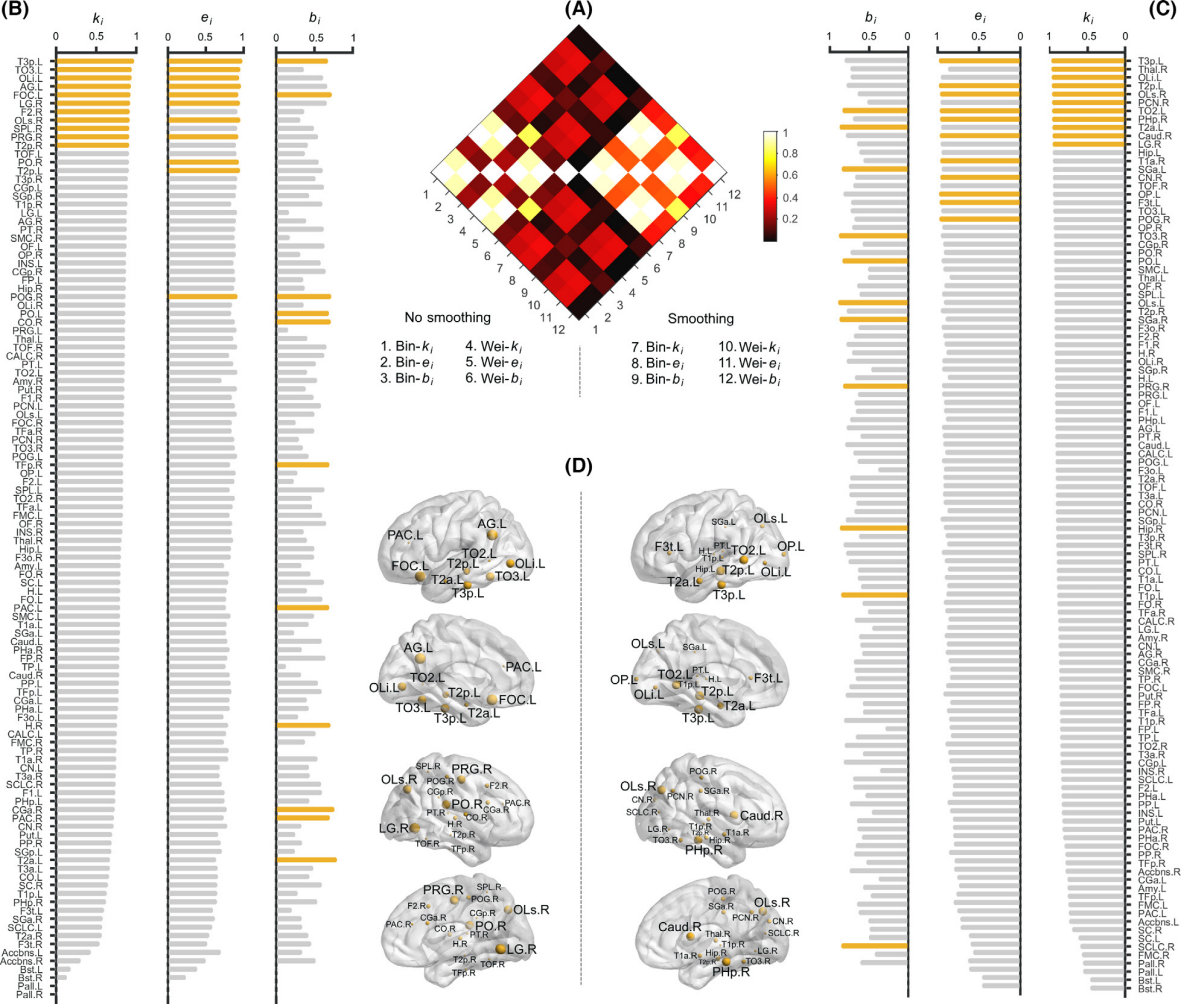
The test–retest (TRT) reliability of nodal centralities under the Harvard‐Oxford atlas (HOA) parcellation scheme. The results were presented in a similar manner to that in Fig. 11. All regional abbreviations can be found in Table S2.

The mean ICC values of nodal centralities under different analytical categories were visualized in Fig. 10. Generally, high TRT reliability was found regardless of nodal centrality metrics and analytical schemes employed (0.741 ± 0.076 overall all factors). To understand nodal reliability more deeply, we also calculated their pairwise spatial correlations across regions and obtained a 12 × 12 correlation matrix under each brain parcellation scheme (Fig. 11A for AAL and Fig. 12A for HOA). Again, we found that the correlation values among nodal reliability maps derived from nonsmoothed data (AAL: 0.688 ± 0.268; HOA: 0.517 ± 0.394) and from smoothed data (AAL: 0.703 ± 0.234; HOA: 0.670 ± 0.271) were significantly larger than those calculated between nonsmoothed and smoothed data (AAL: 0.235 ± 0.113; HOA: 0.157 ± 0.138) (permutation test, 10,000 iterations, *P *<* *0.001 for all comparisons). These findings indicate that, in addition to nodal centralities, spatial smoothing remarkably affects spatial distribution of nodal reliability for morphological brain networks (Fig. 11B vs. Fig. 11C for AAL and Fig. 12B vs. Fig. 12C for HOA). Thus, we used a similar procedure to hub detection to identify regions that consistently exhibited high reliability (top 10%) over nodal centrality metrics and network types but for nonsmoothed and smoothed data (Fig. 11D for AAL and Fig. 12D for HOA). Overall, regions with high reliability were mainly association cortices of temporal, occipital and parietal regions depending on the spatial smoothing and brain parcellation scheme. Table 3 further summarizes the most consistent regions showing high reliability (scored > 3). Notably, the right superior occipital gyrus and middle occipital gyrus under the AAL parcellation and the left posterior division of inferior temporal gyrus and right superior division of lateral occipital cortex under the HOA parcellation were identified for both nonsmoothed and smoothed data. In addition, we compared nodal ICC values among the three centrality metrics (one‐way ANOVA) and found significant main effects (all *P *<* *0.001) driven by higher reliability of nodal degree and efficiency than nodal betweenness. This was independent on choices of spatial smoothing, brain parcellation and network type. Finally, we also compared nodal ICC values between cortical and subcortical regions and consistently found no significant differences regardless of nodal centrality metrics and analytical strategies (all *P *>* *0.05).

**Table 3:** Regions consistently showing high reliabilities

Name	Score	Category	Name	Score	Category
Nonsmo‐AAL			Smo‐AAL		
Precentral gyrus (L)	4	Primary	Superior occipital gyrus (R)	6	Association
Middle frontal gyrus (R)	4	Association	Inferior parietal, but supramarginal and angular gyri (R)	5	Association
Superior occipital gyrus (R)	4	Association	Cuneus (R)	4	Association
Middle occipital gyrus (R)	4	Association	Middle occipital gyrus (R)	4	Association
Angular gyrus (R)	4	Association	Inferior parietal, but supramarginal and angular gyri (L)	4	Association
Middle temporal gyrus (R)	4	Association	Precuneus (R)	4	Association
			Superior temporal gyrus (R)	4	Association
Nonsmo‐HOA			Smo‐HOA		
Angular gyrus (L)	5	Association	Middle temporal gyrus, posterior division (L)	4	Association
Frontal orbital cortex (L)	5	Paralimbic	Middle temporal gyrus, temporooccipital part (L)	4	Association
Lingual gyrus (R)	5	Association	Inferior temporal gyrus, posterior division (L)	4	Association
Parietal operculum cortex (R)	4	Association	Lateral occipital cortex, superior division (R)	4	Association
Precentral gyrus (R)	4	Primary	Parahippocampal gyrus, posterior division (R)	4	Paralimbic
Inferior temporal gyrus, posterior division (L)	4	Association			
Inferior temporal gyrus, temporooccipital part (L)	4	Association			
Lateral occipital cortex, superior division (R)	4	Association			
Lateral occipital cortex, inferior division (L)	4	Association			

Nonsmo, no smoothing; Smo, smoothing; AAL, Anatomical Automatic Labeling atlas; HOA, Harvard‐Oxford atlas; L, left; R, right.

#### Effects of spatial smoothing and network type on TRT reliability of nodal centralities

A two‐way repeated ANOVA was performed for each nodal centrality metric (degree, efficiency and betweenness) under each parcellation scheme (AAL and HOA). The results showed that spatial smoothing and network type significantly modulated nodal reliability in an interactive manner regardless of the brain parcellation schemes and nodal centrality metrics used (all *P *<* *0.05). Post‐hoc comparisons revealed that performing spatial smoothing significantly improved nodal reliability of all the three centrality metrics for both binary and weighted networks but to different extents (all *P *<* *0.001). In addition, binary network analysis outperformed weighted network analysis with respect to reliability of nodal betweenness for both smoothed (*P *=* *0.037 for AAL and <0.001 for HOA) and nonsmoothed data (*P *<* *0.001 for AAL and HOA).

#### Relationship between nodal centrality and ICC

**Figure 13:**
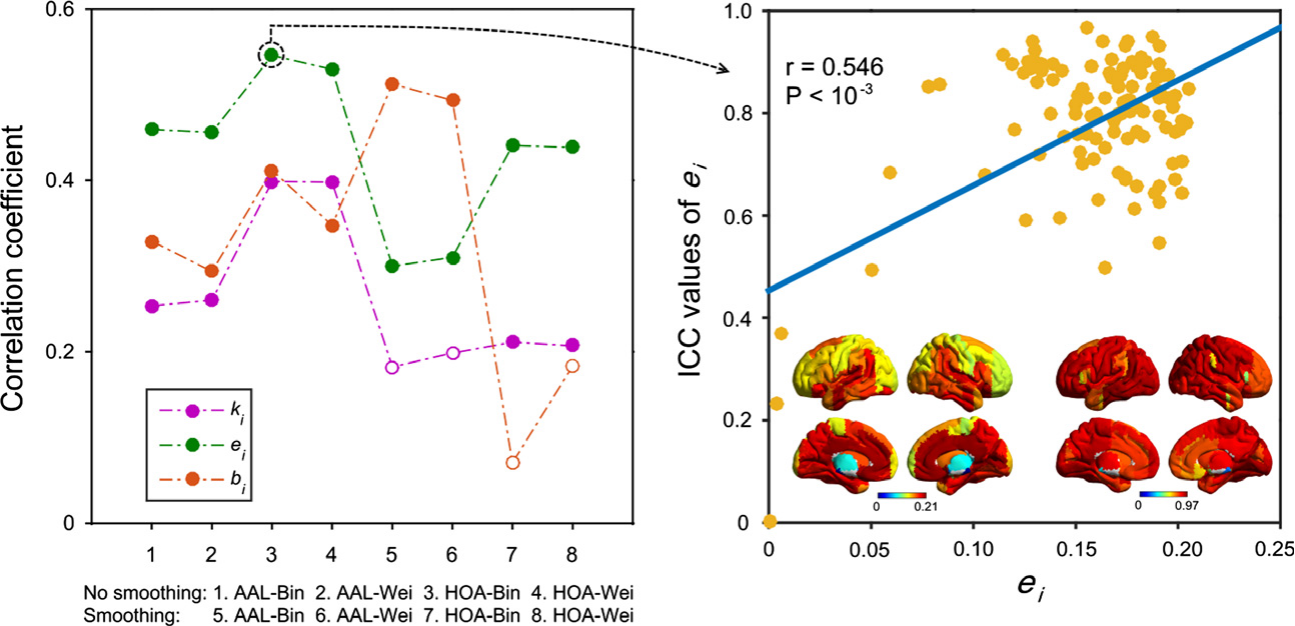
Relationship between nodal centralities and their reliabilities. In most cases, significantly positive correlations (solid circles) were observed between nodal centralities and their corresponding ICC values for each nodal centrality metric in particular for nodal efficiency. ICC, intra‐class correlation; Bin, binary network; Wei, weighted network.

To determine whether nodal ICCs were related to their centralities in morphological brain networks, we calculated the Pearson correlation coefficients across regions between each nodal centrality metric (averaged across participants and data sessions) and their corresponding ICC values under each analytical strategy. Generally, significantly positive correlations were observed that were largely reproducible regardless of the analytical strategies and nodal centrality metrics used (Fig. 13). These results indicate higher reliability for more central nodes in morphological brain networks.

## Discussion

In this study, we constructed KLS‐based, individual‐level, whole‐brain morphological brain networks using structural MRI data and systematically investigated their topological organization under different analytical strategies. We found that the morphological brain networks were specifically organized in a high‐efficient, small‐world and modular manner with several highly connected hubs. Moreover, we demonstrated that these configurations of morphological brain networks were dependent on different choices of spatial smoothing, brain parcellation and network type. Further examination of long‐term TRT reliability showed that all the topological properties had fair to excellent reliability but spatial smoothing significantly improved reliability and nodal degree and efficiency outperformed nodal betweenness. Interestingly, nodal centralities were positively correlated with their ICC values, suggesting higher reliability for more central nodes. Taken together, these findings suggest that KLS‐based, single‐subject morphological brain network is a meaningful and reliable method in characterizing the brain organization and thus opens a new avenue toward understanding structural substrate of intersubject variability in behavior and function.

### KLS‐based morphological brain networks

Currently, morphological brain networks are mainly derived by estimating interregional correlations in morphological features (e.g., cortical thickness or GM volume) at either group (He et al. 2007; Bassett et al. 2008) or individual (Tijms et al. 2012; Batalle et al. 2013) level. These correlation‐based methods require the normal distribution of morphological features across subjects or spatial locations or voxels. In contrast, the KLS‐based method used here has no such restriction, therefore may be more suitable for studying the human brain particularly given its complex folding structure. Moreover, based on empirical data, several recent studies have demonstrated the effectiveness of the KL/KLS method in studying the human brain in different populations (Velazquez and Galan 2013; Kong et al. 2014, 2015). Additionally, compared with a recently proposed single‐subject morphological network method (Tijms et al. 2012), the KLS‐based method has distinct advantages in network node definition which allows flexible choices of brain parcellation without any restriction on regional size and shape. This allows researchers to freely generate study‐specific, customized brain atlas according to their study objectives. All these features suggest that KLS‐based morphological network analysis could serve as a promising method to study morphological organization of the human brain at an individual level.

### Specifically organized, analytical strategy‐dependent and long‐term reliable morphological brain networks

We showed that the KLS‐based, single‐subject morphological brain networks globally exhibited high‐efficient, small‐world and modular architecture. These findings are consistent with previous morphological brain network studies (He et al. 2007; Bassett et al. 2008; Chen et al. 2008; Tijms et al. 2012). Currently, the human brain is universally believed to have evolved to support both specialized or modular processing in local regions and distributed or integrated processing over the entire brain (Sporns et al. 2004; Bullmore and Sporns 2009, 2012; Meunier et al. 2009; He and Evans 2010). Here, our findings provide further empirical evidence to support the theory that the human cortical morphology has evolved into a complex but efficient neuronal architecture which confers an optimal balance between local specialization and global integration to maximize the power of parallel information processing. Locally, nodal centrality analysis of morphological brain networks revealed a heterogeneous distribution over the brain with several association and paralimbic regions (e.g., the precuneus, angular gyrus, cingulate gyrus, and parahippocampal gyrus) topologically holding central positions. These regions are largely comparable with the putative hubs reported in previous morphological, structural and functional brain networks (Achard et al. 2006; He et al. 2007, 2009b; Hagmann et al. 2008; Buckner et al. 2009; Gong et al. 2009; Tomasi and Volkow 2010). Recently, identifying brain hubs and further studying their vulnerability in various brain disorders are becoming a hot research topic (van den Heuvel and Sporns 2013; Crossley et al. 2014). Therefore, the current work provides an alternative method for researchers to explore hubs of the brain under both healthy and pathological conditions. Overall, these findings suggest that KLS‐based, single‐subject morphological brain networks are wired in an organized manner rather than randomly connected. Particularly, our global and local findings are largely consistent with those reported in a recent study which also investigated the topological organization of individual‐level morphological brain networks based on the KLS‐based method (Kong et al. 2015). These findings suggest a stable intrinsic architecture of KLS‐based morphological brain networks.

Nonetheless, compared with what was done by Kong and colleagues, the current work addressed several key questions which are important before the practical application of the KLS‐based approach. We first examined long‐term TRT reliability of KLS‐based morphological brain networks based on datasets collected with an interval of approximate 6 weeks. This is in contrast with what was done by Kong et al. (2015) who used datasets acquired on the same day to examine short‐term reliability. Long‐term reliability analysis is particularly important for newly developed methods before their clinical applications to identify stable biomarkers. We showed that most global and nodal network measures exhibited fair to excellent long‐term TRT reliability. Together with the high short‐term TRT reliability as reported in Kong et al. (2015), we propose the KLS‐based single‐subject morphological brain network analysis as a stable approach for future research to study effects of interest, such as topological reorganization in various brain disorders. Furthermore, we evaluated the effects of several factors on topological organization of KLS‐based morphological brain networks, including network type, brain parcellation and spatial smoothing. Insights into these issues are vital for researchers to determine their analytical strategies when using this approach. We showed that quantitative characterization of KLS‐based morphological brain networks was significantly affected by different analytical strategies. For example, spatial smoothing remarkably modulated spatial distribution of nodal centrality. The dependence of morphological brain networks on choices of preprocessing and network construction methods is consistent with previous brain network studies based on other neuroimaging modalities (Wang et al. 2009; Cole et al. 2010; Zalesky et al. 2010; Liang et al. 2012). Thus, the current results together with previous findings collectively highlight that for imaging connectomics studies, researchers should carefully choose their analytical pipelines and explain the findings when results are compared across studies with different processing methods. Apart from numerical values, the TRT reliability analysis further showed that different analytical strategies also affected the stability of KLS‐based morphological brain networks, consistent with previous brain network studies (Bassett et al. 2011; Wang et al. 2011; Liang et al. 2012; Buchanan et al. 2014; Zhao et al. 2015). Particularly, performing spatial smoothing significantly improved the reliability of both global and nodal network properties. This could be due to the elevated correspondence of brain structures among participants. To test this interpretation, we computed Pearson correlations in the KLS matrices across matrix elements for any pair of participants before and after spatial smoothing and found significantly higher intersubject similarities when spatial smoothing was conducted (*P *<* *0.001 for both AAL and HOA brain parcellations). In addition, we found that nodal degree and efficiency outperformed nodal betweenness with respect to reliability. The higher reliability of nodal degree is consistent with our previous functional brain network study (Wang et al. 2011), possibly due to its conciseness and directness in definition. These findings provide important guidance on how to choose reliable analytical strategies and network metrics for KLS‐based, single‐subject morphological brain networks. Finally, we noted that several association and paralimbic regions were consistently identified as hubs (e.g., the precuneus, angular gyrus, parahippocampal gyrus, temporal and lateral occipital cortex) and consistently exhibited high reliability. Further correlation analysis revealed positive correlations between nodal centralities and their ICC values, suggesting higher reliability for more central regions of morphological brain networks. This is consistent with our previous finding of functional brain networks (Wang et al. 2011). These findings collectively imply that hub architecture is a stable organizational principle for the human brain networks (Buckner et al. 2009). Given the fact that brain hubs are generally implicated in various brain disorders (Crossley et al. 2014; Stam 2014), the high reliability of hubs makes them potential candidates to serve as reliable markers for disease diagnosis and prognosis, an interesting research topic in the future.

Taken together, the KLS‐based, single‐subject morphological brain networks are specifically organized, analytical strategy sensitive and TRT reliable, therefore opening a new avenue in linking morphological network variability and interindividual differences in behavior and cognition, although more studies are needed to elucidate the underlying biological significance.

### Possible biological interpretation of KLS‐based morphological brain networks

In the current study, we calculated interregional similarity (as quantified by the KLS) in their distributions of GM volume to define morphological connectivity. However, the biological meaning underlying the similarity is not clear (Kong et al. 2014). Nevertheless, it should be noted that interregional covariance in morphological features has been observed as early as 1997 for several components of the human visual system (Andrews et al. 1997). Not limited to the visual system, the morphological coordination is demonstrated to expand to the whole brain, forming a morphological covariance network (He et al. 2007; Bassett et al. 2008). Moreover, an increasing number of studies have shown that the morphological covariance networks exhibit adaptive reorganization during normal development (Zielinski et al. 2010; Fan et al. 2011; Alexander‐Bloch et al. 2013b) and aging (Chen et al. 2011; Wu et al. 2012; Zhu et al. 2012) and in various brain disorders (He et al. 2008, 2009a; Seeley et al. 2009; Zhang et al. 2012). These studies jointly suggest that morphological covariance networks are biological meaningful in capturing potential mechanisms involved in these processes. Although the biological significance of morphological covariance is still not fully understood, accumulating evidence indicates that heredity, experience‐related plasticity, mutually trophic influences or coordinated neurodevelopment and aging trajectories play important roles in the formation morphological brain networks [for recent reviews, see (Alexander‐Bloch et al. 2013a; Evans 2013)]. In addition, there is another possible explanation coming from the axonal extension theory (Van Essen 1997), which suggests that connected areas tend to be pulled together by a tension from the axons between them. Therefore, it is plausible to speculate that these factors may also contribute to KLS‐based, individual‐level morphological brain networks studied here. Insights into this speculation could benefit from future studies by linking morphological brain organization with behavior performances or cognitive abilities for the same cohort of participants, or examining genetic, developmental, training‐induced, or brain disorder‐related changes of KLS‐based single‐subject morphological brain networks. Of note, the KLS‐based approach has been demonstrated to be capable of capturing age‐related changes in morphological brain networks (Kong et al. 2015).

## Limitations and Future Directions

First, this study only examined several most prevalent topological attributes for single‐subject morphological brain networks. Besides these features, there are many other topological properties that are consistently observed in human brain networks, such as the “rich‐club” organization (van den Heuvel and Sporns 2011), hierarchy (Bassett et al. 2008) and heavy‐tailed degree distribution (Newman 2003; Avena‐Koenigsberger et al. 2015; Roberts et al. 2015). Future studies are needed to determine whether these configurations hold in KLS‐based, single‐subject morphological brain networks. Second, in the current study, we employed the AAL and HOA, two widely used atlases in previous brain network studies, to examine the effects of different brain parcellation schemes on topological organization of KLS‐based individual‐level morphological brain networks. Nevertheless, it should be noted that how to divide the brain into different ROIs to define network nodes is still an open question (de Reus and van den Heuvel 2013). Apart from the two atlases studied here, there are many other brain atlases available, such as the Automated Non‐linear Image Matching and Anatomical Labeling algorithm (Collins et al. 1995) and the LONI Probabilistic Brain Atlas (LPBA40) (Shattuck et al. 2008). Particularly, the Freesurfer provides a classification technique for automatically labeling individual brains into different regions that is robust to intersubject anatomical variability (Fischl et al. 2004; Desikan et al. 2006). In the future, it is important to compare these atlases to provide more comprehensive insights into how different parcellation schemes affect topological organization of individual‐level morphological brain networks. In addition, most of the current brain atlases are comparable with respect to the number of brain regions (i.e., similar spatial scale). Future studies are also needed to investigate how individual‐level morphological brain networks topologically organize at different scales by employing atlases that span several orders of magnitude in the number of regions or using an iterative random parcellation method (Fornito et al. 2010; Zalesky et al. 2010). Third, based on data from healthy participants we demonstrated that the KLS‐based method was reproducible and reliable in characterizing single‐subject morphological brain networks. The next step is to examine whether this method could reveal sensitive and reliable biomarkers associated with different states and various brain disorders. Third, single‐subject morphological brain networks were constructed by calculating interregional similarities in regional GM volume in the current study. Straightforwardly, this method could be extended to other morphological features (e.g., cortical thickness) or even images of other modalities (e.g., positron emission tomography). Furthermore, in addition to the KLS measure used here, there are also other measures to quantify the similarity of two curves, such as the Bhattacharyya distance (Zhou and Chellappa 2006) or related measures (De Maesschalck et al. 2000; Comaniciu et al. 2003). Future studies are required to examine unique insights into morphological brain networks from different choices of these factors. Finally, an increasing number of studies have examined the relationship between anatomical and functional brain networks and find that functional connectivity profiles are largely shaped but not fully determined by structural pathways (Wang et al. 2015b). In this regard, it is an interesting topic for future research to determine the similarities and differences between single‐subject morphological brain networks and those derived from other neuroimaging modalities in mapping and characterizing the human connectome.

## Conclusion

This study demonstrates that KLS‐based, single‐subject morphological brain networks are specifically organized with several nontrivial topological features, which are TRT reliable but depend on choices of analytical strategies. This method therefore could complement the current methodology of neuroimaging connectomics and open a new avenue toward understanding structural substrate of intersubject variability in behavior and function from a network perspective.

## Conflict of Interest

The authors declare that there are no conflicts of interest.

## Supporting information


**Table S1**. Regions of interest from the AAL atlas.
**Table S2.** Regions of interest from the HOA atlas.

## References

[brb3448-bib-0001] Achard, S. , R. Salvador , B. Whitcher , J. Suckling , and E. Bullmore . 2006 A resilient, low‐frequency, small‐world human brain functional network with highly connected association cortical hubs. J. Neurosci. 26:63–72. 16399673 10.1523/JNEUROSCI.3874-05.2006 PMC6674299

[brb3448-bib-0002] Alexander‐Bloch, A. , J. N. Giedd , and E. Bullmore . 2013a Imaging structural co‐variance between human brain regions. Nat. Rev. Neurosci. 14:322–336. 23531697 10.1038/nrn3465 PMC4043276

[brb3448-bib-0003] Alexander‐Bloch, A. , A. Raznahan , E. Bullmore , and J. Giedd . 2013b The convergence of maturational change and structural covariance in human cortical networks. J. Neurosci. 33:2889–2899. 23407947 10.1523/JNEUROSCI.3554-12.2013 PMC3711653

[brb3448-bib-0004] Alt, H. , and M. Godau . 1995 Computing the Fréchet distance between two polygonal curves. Int. J. Comput. Geom. Appl. 5:75–91.

[brb3448-bib-0005] Andrews, T. J. , S. D. Halpern , and D. Purves . 1997 Correlated size variations in human visual cortex, lateral geniculate nucleus, and optic tract. J. Neurosci. 17:2859–2868. 9092607 10.1523/JNEUROSCI.17-08-02859.1997 PMC6573115

[brb3448-bib-0006] Ashburner, J. 2007 A fast diffeomorphic image registration algorithm. NeuroImage 38:95–113. 17761438 10.1016/j.neuroimage.2007.07.007

[brb3448-bib-0007] Avena‐Koenigsberger, A. , J. Goni , R. Sole , and O. Sporns . 2015 Network morphospace. J. R. Soc. Interface 12:20140881. 25540237 10.1098/rsif.2014.0881 PMC4305402

[brb3448-bib-0008] Barch, D. M. 2013 Brain network interactions in health and disease. Trends Cogn. Sci. 17:603–605. 24080424 10.1016/j.tics.2013.09.004 PMC3858486

[brb3448-bib-0009] Bassett, D. S. , E. Bullmore , B. A. Verchinski , V. S. Mattay , D. R. Weinberger , and A. Meyer‐Lindenberg . 2008 Hierarchical organization of human cortical networks in health and schizophrenia. J. Neurosci. 28:9239–9248. 18784304 10.1523/JNEUROSCI.1929-08.2008 PMC2878961

[brb3448-bib-0010] Bassett, D. S. , J. A. Brown , V. Deshpande , J. M. Carlson , and S. T. Grafton . 2011 Conserved and variable architecture of human white matter connectivity. NeuroImage 54:1262–1279. 20850551 10.1016/j.neuroimage.2010.09.006

[brb3448-bib-0011] Batalle, D. , E. Munoz‐Moreno , F. Figueras , N. Bargallo , E. Eixarch , and E. Gratacos . 2013 Normalization of similarity‐based individual brain networks from gray matter MRI and its association with neurodevelopment in infants with intrauterine growth restriction. NeuroImage 83:901–911. 23886985 10.1016/j.neuroimage.2013.07.045

[brb3448-bib-0012] Biswal, B. , F. Z. Yetkin , V. M. Haughton , and J. S. Hyde . 1995 Functional connectivity in the motor cortex of resting human brain using echo‐planar MRI. Magn. Reson. Med. 34:537–541. 8524021 10.1002/mrm.1910340409

[brb3448-bib-0013] Biswal, B. B. , M. Mennes , X. N. Zuo , S. Gohel , C. Kelly , S. M. Smith , et al. 2010 Toward discovery science of human brain function. Proc. Natl Acad. Sci. USA 107:4734–4739. 20176931 10.1073/pnas.0911855107 PMC2842060

[brb3448-bib-0014] Borsboom, D. , and A. O. Cramer . 2013 Network analysis: an integrative approach to the structure of psychopathology. Annu. Rev. Clin. Psychol. 9:91–121. 23537483 10.1146/annurev-clinpsy-050212-185608

[brb3448-bib-0015] Botev, Z. I. , J. F. Grotowski , and D. P. Kroese . 2010 Kernel density estimation via diffusion. Ann. Stat. 38:2916–2957.

[brb3448-bib-0016] Buchanan, C. R. , C. R. Pernet , K. J. Gorgolewski , A. J. Storkey , and M. E. Bastin . 2014 Test–retest reliability of structural brain networks from diffusion MRI. NeuroImage 86:231–243. 24096127 10.1016/j.neuroimage.2013.09.054

[brb3448-bib-0017] Buckner, R. L. , J. Sepulcre , T. Talukdar , F. M. Krienen , H. Liu , T. Hedden , et al. 2009 Cortical hubs revealed by intrinsic functional connectivity: mapping, assessment of stability, and relation to Alzheimer's disease. J. Neurosci. 29:1860–1873. 19211893 10.1523/JNEUROSCI.5062-08.2009 PMC2750039

[brb3448-bib-0018] Bullmore, E. T. , and D. S. Bassett . 2011 Brain graphs: graphical models of the human brain connectome. Annu. Rev. Clin. Psychol. 7:113–140. 21128784 10.1146/annurev-clinpsy-040510-143934

[brb3448-bib-0019] Bullmore, E. , and O. Sporns . 2009 Complex brain networks: graph theoretical analysis of structural and functional systems. Nat. Rev. Neurosci. 10:186–198. 19190637 10.1038/nrn2575

[brb3448-bib-0020] Bullmore, E. , and O. Sporns . 2012 The economy of brain network organization. Nat. Rev. Neurosci. 13:336–349. 22498897 10.1038/nrn3214

[brb3448-bib-0021] Burnham, K. P. , and D. R. Anderson . 2002 Model selection and multimodel inference: a practical information‐theoretic approach. Springer Science & Business Media, New York.

[brb3448-bib-0022] Chen, Z. J. , Y. He , P. Rosa , J. Germann , and A. C. Evans . 2008 Revealing modular architecture of human brain structural networks by using cortical thickness from MRI. Cereb. Cortex 18:2374–2381. 18267952 10.1093/cercor/bhn003 PMC2733312

[brb3448-bib-0023] Chen, Z. J. , Y. He , P. Rosa‐Neto , G. L. Gong , and A. C. Evans . 2011 Age‐related alterations in the modular organization of structural cortical network by using cortical thickness from MRI. NeuroImage 56:235–245. 21238595 10.1016/j.neuroimage.2011.01.010

[brb3448-bib-0024] Cole, M. W. , S. Pathak , and W. Schneider . 2010 Identifying the brain's most globally connected regions. NeuroImage 49:3132–3148. 19909818 10.1016/j.neuroimage.2009.11.001

[brb3448-bib-0025] Cole, M. W. , D. S. Bassett , J. D. Power , T. S. Braver , and S. E. Petersen . 2014 Intrinsic and task‐evoked network architectures of the human brain. Neuron 83:238–251. 24991964 10.1016/j.neuron.2014.05.014 PMC4082806

[brb3448-bib-0026] Collins, D. L. , C. J. Holmes , T. M. Peters , and A. C. Evans . 1995 Automatic 3‐D model‐based neuroanatomical segmentation. Hum. Brain Mapp. 3:190–208.

[brb3448-bib-0027] Comaniciu, D. , V. Ramesh , and P. Meer . 2003 Kernel‐based object tracking. IEEE Trans. Pattern. Anal. Mach. Intell. 25:564–577.

[brb3448-bib-0028] Craddock, R. C. , S. Jbabdi , C. G. Yan , J. T. Vogelstein , F. X. Castellanos , A. Di Martino , et al. 2013 Imaging human connectomes at the macroscale. Nat. Methods 10:524–539. 23722212 10.1038/nmeth.2482 PMC4096321

[brb3448-bib-0029] Crossley, N. A. , A. Mechelli , J. Scott , F. Carletti , P. T. Fox , P. McGuire , et al. 2014 The hubs of the human connectome are generally implicated in the anatomy of brain disorders. Brain 137:2382–2395. 25057133 10.1093/brain/awu132 PMC4107735

[brb3448-bib-0030] De Maesschalck, R. , D. Jouan‐Rimbaud , and D. L. Massart . 2000 The mahalanobis distance. Chemometr. Intell. Lab. Syst. 50:1–18.

[brb3448-bib-0031] Desikan, R. S. , F. Ségonne , B. Fischl , B. T. Quinn , B. C. Dickerson , D. Blacker , et al. 2006 An automated labeling system for subdividing the human cerebral cortex on MRI scans into gyral based regions of interest. NeuroImage 31:968–980. 16530430 10.1016/j.neuroimage.2006.01.021

[brb3448-bib-0032] Evans, A. C. 2013 Networks of anatomical covariance. NeuroImage 80:489–504. 23711536 10.1016/j.neuroimage.2013.05.054

[brb3448-bib-0033] Fan, Y. , F. Shi , J. K. Smith , W. Lin , J. H. Gilmore , and D. Shen . 2011 Brain anatomical networks in early human brain development. NeuroImage 54:1862–1871. 20650319 10.1016/j.neuroimage.2010.07.025 PMC3023885

[brb3448-bib-0034] Fischl, B. , A. van der Kouwe , C. Destrieux , E. Halgren , F. Ségonne , D. H. Salat , et al. 2004 Automatically parcellating the human cerebral cortex. Cereb. Cortex 14:11–22. 14654453 10.1093/cercor/bhg087

[brb3448-bib-0035] Fornito, A. , and E. T. Bullmore . 2015 Connectomics: a new paradigm for understanding brain disease. Eur. Neuropsychopharmacol. 25:733–748. 24726580 10.1016/j.euroneuro.2014.02.011

[brb3448-bib-0036] Fornito, A. , A. Zalesky , and E. T. Bullmore . 2010 Network scaling effects in graph analytic studies of human resting‐state FMRI data. Front. Syst. Neurosci. 4:22. 20592949 10.3389/fnsys.2010.00022 PMC2893703

[brb3448-bib-0037] Gong, G. , Y. He , L. Concha , C. Lebel , D. W. Gross , A. C. Evans , et al. 2009 Mapping anatomical connectivity patterns of human cerebral cortex using in vivo diffusion tensor imaging tractography. Cereb. Cortex 19:524–536. 18567609 10.1093/cercor/bhn102 PMC2722790

[brb3448-bib-0038] Hagmann, P. , M. Kurant , X. Gigandet , P. Thiran , V. J. Wedeen , R. Meuli , et al. 2007 Mapping human whole‐brain structural networks with diffusion MRI. PLoS One 2:e597. 17611629 10.1371/journal.pone.0000597 PMC1895920

[brb3448-bib-0039] Hagmann, P. , L. Cammoun , X. Gigandet , R. Meuli , C. J. Honey , V. J. Wedeen , et al. 2008 Mapping the structural core of human cerebral cortex. PLoS Biol. 6:e159. 18597554 10.1371/journal.pbio.0060159 PMC2443193

[brb3448-bib-0040] He, Y. , and A. Evans . 2010 Graph theoretical modeling of brain connectivity. Curr. Opin. Neurol. 23:341–350. 20581686 10.1097/WCO.0b013e32833aa567

[brb3448-bib-0041] He, Y. , Z. J. Chen , and A. C. Evans . 2007 Small‐world anatomical networks in the human brain revealed by cortical thickness from MRI. Cereb. Cortex 17:2407–2419. 17204824 10.1093/cercor/bhl149

[brb3448-bib-0042] He, Y. , Z. Chen , and A. Evans . 2008 Structural insights into aberrant topological patterns of large‐scale cortical networks in Alzheimer's disease. J. Neurosci. 28:4756–4766. 18448652 10.1523/JNEUROSCI.0141-08.2008 PMC6670444

[brb3448-bib-0043] He, Y. , A. Dagher , Z. Chen , A. Charil , A. Zijdenbos , K. Worsley , et al. 2009a Impaired small‐world efficiency in structural cortical networks in multiple sclerosis associated with white matter lesion load. Brain 132:3366–3379. 19439423 10.1093/brain/awp089 PMC2792366

[brb3448-bib-0044] He, Y. , J. Wang , L. Wang , Z. J. Chen , C. Yan , H. Yang , et al. 2009b Uncovering intrinsic modular organization of spontaneous brain activity in humans. PLoS One 4:e5226. 19381298 10.1371/journal.pone.0005226 PMC2668183

[brb3448-bib-0045] van den Heuvel, M. P. , and O. Sporns . 2011 Rich‐club organization of the human connectome. J. Neurosci. 31:15775–15786. 22049421 10.1523/JNEUROSCI.3539-11.2011 PMC6623027

[brb3448-bib-0046] van den Heuvel, M. P. , and O. Sporns . 2013 Network hubs in the human brain. Trends Cogn. Sci. 17:683–696. 24231140 10.1016/j.tics.2013.09.012

[brb3448-bib-0047] Hilgetag, C. C. , and H. Barbas . 2005 Developmental mechanics of the primate cerebral cortex. Anat. Embryol. (Berl) 210:411–417. 16175385 10.1007/s00429-005-0041-5

[brb3448-bib-0048] Iturria‐Medina, Y. , E. J. Canales‐Rodriguez , L. Melie‐Garcia , P. A. Valdes‐Hernandez , E. Martinez‐Montes , Y. Aleman‐Gomez , et al. 2007 Characterizing brain anatomical connections using diffusion weighted MRI and graph theory. NeuroImage 36:645–660. 17466539 10.1016/j.neuroimage.2007.02.012

[brb3448-bib-0049] Kelly, C. , B. B. Biswal , R. C. Craddock , F. X. Castellanos , and M. P. Milham . 2012 Characterizing variation in the functional connectome: promise and pitfalls. Trends Cogn Sci. 16:181–188. 22341211 10.1016/j.tics.2012.02.001 PMC3882689

[brb3448-bib-0050] Kennedy, D. N. , N. Lange , N. Makris , J. Bates , J. Meyer , and V. S. Jr Caviness . 1998 Gyri of the human neocortex: an MRI‐based analysis of volume and variance. Cereb. Cortex 8:372–384. 9651132 10.1093/cercor/8.4.372

[brb3448-bib-0051] Kong, X. Z. , X. Wang , L. Huang , Y. Pu , Z. Yang , X. Dang , et al. 2014 Measuring individual morphological relationship of cortical regions. J. Neurosci. Methods 237:103–107. 25220868 10.1016/j.jneumeth.2014.09.003

[brb3448-bib-0052] Kong, X. Z. , Z. Liu , L. Huang , X. Wang , Z. Yang , G. Zhou , et al. 2015 Mapping individual brain networks using statistical similarity in regional morphology from MRI. PLoS One 10:e0141840. 26536598 10.1371/journal.pone.0141840 PMC4633111

[brb3448-bib-0053] Kullback, S. , and R. A. Leibler . 1951 On information and sufficiency. Ann. Math. Stat. 22:79–86.

[brb3448-bib-0054] Liang, X. , J. Wang , C. Yan , N. Shu , K. Xu , G. Gong , et al. 2012 Effects of different correlation metrics and preprocessing factors on small‐world brain functional networks: a resting‐state functional MRI study. PLoS One 7:e32766. 22412922 10.1371/journal.pone.0032766 PMC3295769

[brb3448-bib-0055] Makris, N. , J. W. Meyer , J. F. Bates , E. H. Yeterian , D. N. Kennedy , and V. S. Caviness . 1999 MRI‐Based topographic parcellation of human cerebral white matter and nuclei II. Rationale and applications with systematics of cerebral connectivity. NeuroImage 9:18–45. 9918726 10.1006/nimg.1998.0384

[brb3448-bib-0056] Maslov, S. , and K. Sneppen . 2002 Specificity and stability in topology of protein networks. Science 296:910–913. 11988575 10.1126/science.1065103

[brb3448-bib-0057] Meunier, D. , S. Achard , A. Morcom , and E. Bullmore . 2009 Age‐related changes in modular organization of human brain functional networks. NeuroImage 44:715–723. 19027073 10.1016/j.neuroimage.2008.09.062

[brb3448-bib-0058] Newman, M. E. J. 2003 The structure and function of complex networks. SIAM Rev. 45:167–256.

[brb3448-bib-0059] Parzen, E. . 1962 On estimation of a probability density function and mode. Ann. Math. Stat. 33:1065–1076.

[brb3448-bib-0060] Raj, A. , S. G. Mueller , K. Young , K. D. Laxer , and M. Weiner . 2010 Network‐level analysis of cortical thickness of the epileptic brain. NeuroImage 52:1302–1313. 20553893 10.1016/j.neuroimage.2010.05.045 PMC2910126

[brb3448-bib-0061] de Reus, M. A. , and M. P. van den Heuvel . 2013 The parcellation‐based connectome: limitations and extensions. NeuroImage 80:397–404. 23558097 10.1016/j.neuroimage.2013.03.053

[brb3448-bib-0062] Roberts, J. A. , T. W. Boonstra , and M. Breakspear . 2015 The heavy tail of the human brain. Curr. Opin. Neurobiol. 31:164–172. 25460073 10.1016/j.conb.2014.10.014

[brb3448-bib-0063] Rosenblatt, M. 1956 Remarks on some nonparametric estimates of a density function. Ann. Math. Stat. 27:832–837.

[brb3448-bib-0064] Rubinov, M. , and O. Sporns . 2010 Complex network measures of brain connectivity: uses and interpretations. NeuroImage 52:1059–1069. 19819337 10.1016/j.neuroimage.2009.10.003

[brb3448-bib-0065] Salvador, R. , J. Suckling , M. R. Coleman , J. D. Pickard , D. Menon , and E. Bullmore . 2005 Neurophysiological architecture of functional magnetic resonance images of human brain. Cereb. Cortex 15:1332–1342. 15635061 10.1093/cercor/bhi016

[brb3448-bib-0066] Sanabria‐Diaz, G. , L. Melie‐Garcia , Y. Iturria‐Medina , Y. Aleman‐Gomez , G. Hernandez‐Gonzalez , L. Valdes‐Urrutia , et al. 2010 Surface area and cortical thickness descriptors reveal different attributes of the structural human brain networks. NeuroImage 50:1497–1510. 20083210 10.1016/j.neuroimage.2010.01.028

[brb3448-bib-0067] Seeley, W. W. , R. K. Crawford , J. Zhou , B. L. Miller , and M. D. Greicius . 2009 Neurodegenerative diseases target large‐scale human brain networks. Neuron 62:42–52. 19376066 10.1016/j.neuron.2009.03.024 PMC2691647

[brb3448-bib-0068] Shattuck, D. W. , M. Mirza , V. Adisetiyo , C. Hojatkashani , G. Salamon , K. L. Narr , et al. 2008 Construction of a 3D probabilistic atlas of human cortical structures. NeuroImage 39:1064–1080. 18037310 10.1016/j.neuroimage.2007.09.031 PMC2757616

[brb3448-bib-0069] Shrout, P. E. , and J. L. Fleiss . 1979 Intraclass correlations: uses in assessing rater reliability. Psychol. Bull. 86:420–428. 18839484 10.1037//0033-2909.86.2.420

[brb3448-bib-0070] Sporns, O. 2013 The human connectome: origins and challenges. NeuroImage 80:53–61. 23528922 10.1016/j.neuroimage.2013.03.023

[brb3448-bib-0071] Sporns, O. 2014 Contributions and challenges for network models in cognitive neuroscience. Nat. Neurosci. 17:652–660. 24686784 10.1038/nn.3690

[brb3448-bib-0072] Sporns, O. , D. R. Chialvo , M. Kaiser , and C. C. Hilgetag . 2004 Organization, development and function of complex brain networks. Trends Cogn. Sci. 8:418–425. 15350243 10.1016/j.tics.2004.07.008

[brb3448-bib-0073] Sporns, O. , G. Tononi , and R. Kotter . 2005 The human connectome: a structural description of the human brain. PLoS Comput. Biol. 1:e42. 16201007 10.1371/journal.pcbi.0010042 PMC1239902

[brb3448-bib-0074] Stam, C. J. 2014 Modern network science of neurological disorders. Nat. Rev. Neurosci. 15:683–695. 25186238 10.1038/nrn3801

[brb3448-bib-0075] Tijms, B. M. , P. Series , D. J. Willshaw , and S. M. Lawrie . 2012 Similarity‐based extraction of individual networks from gray matter MRI scans. Cereb. Cortex 22:1530–1541. 21878484 10.1093/cercor/bhr221

[brb3448-bib-0076] Tomasi, D. , and N. D. Volkow . 2010 Functional connectivity density mapping. Proc. Natl Acad. Sci. USA 107:9885–9890. 20457896 10.1073/pnas.1001414107 PMC2906909

[brb3448-bib-0077] Tzourio‐Mazoyer, N. , B. Landeau , D. Papathanassiou , F. Crivello , O. Etard , N. Delcroix , et al. 2002 Automated anatomical labeling of activations in SPM using a macroscopic anatomical parcellation of the MNI MRI single‐subject brain. NeuroImage 15:273–289. 11771995 10.1006/nimg.2001.0978

[brb3448-bib-0078] Van Essen, D. C. 1997 A tension‐based theory of morphogenesis and compact wiring in the central nervous system. Nature 385:313–318. 9002514 10.1038/385313a0

[brb3448-bib-0080] Velazquez, J. L. P. , and R. F. Galan . 2013 Information gain in the brain's resting state: a new perspective on autism. Front. Neuroinform. 7:37. 24399963 10.3389/fninf.2013.00037 PMC3870924

[brb3448-bib-0081] Vertes, P. E. , and E. T. Bullmore . 2015 Annual research review: growth connectomics–the organization and reorganization of brain networks during normal and abnormal development. J. Child Psychol. Psychiatry 56:299–320. 25441756 10.1111/jcpp.12365 PMC4359009

[brb3448-bib-0082] Wang, J. , L. Wang , Y. Zang , H. Yang , H. Tang , Q. Gong , et al. 2009 Parcellation‐dependent small‐world brain functional networks: a resting‐state fMRI study. Hum. Brain Mapp. 30:1511–1523. 18649353 10.1002/hbm.20623 PMC6870680

[brb3448-bib-0083] Wang, J. H. , X. N. Zuo , S. Gohel , M. P. Milham , B. B. Biswal , and Y. He . 2011 Graph theoretical analysis of functional brain networks: test‐retest evaluation on short‐ and long‐term resting‐state functional MRI data. PLoS One 6:e21976. 21818285 10.1371/journal.pone.0021976 PMC3139595

[brb3448-bib-0084] Wang, J. , X. Wang , M. Xia , X. Liao , A. Evans , and Y. He . 2015a GRETNA: a graph theoretical network analysis toolbox for imaging connectomics. Front. Hum. Neurosci. 9:386. 26175682 10.3389/fnhum.2015.00386 PMC4485071

[brb3448-bib-0085] Wang, Z. , Z. Dai , G. Gong , C. Zhou , and Y. He . 2015b Understanding structural‐functional relationships in the human brain: a large‐scale network perspective. Neuroscientist 21:290–305. 24962094 10.1177/1073858414537560

[brb3448-bib-0086] Watts, D. J. , and S. H. Strogatz . 1998 Collective dynamics of ‘small‐world'networks. Nature 393:440–442. 9623998 10.1038/30918

[brb3448-bib-0087] Wu, K. , Y. Taki , K. Sato , S. Kinomura , R. Goto , K. Okada , et al. 2012 Age‐related changes in topological organization of structural brain networks in healthy individuals. Hum. Brain Mapp. 33:552–568. 21391279 10.1002/hbm.21232 PMC6870030

[brb3448-bib-0088] Zalesky, A. , A. Fornito , I. H. Harding , L. Cocchi , M. Yucel , C. Pantelis , et al. 2010 Whole‐brain anatomical networks: does the choice of nodes matter? NeuroImage 50:970–983. 20035887 10.1016/j.neuroimage.2009.12.027

[brb3448-bib-0089] Zhang, Y. , L. Lin , C. P. Lin , Y. Zhou , K. H. Chou , C. Y. Lo , et al. 2012 Abnormal topological organization of structural brain networks in schizophrenia. Schizophr. Res. 141:109–118. 22981811 10.1016/j.schres.2012.08.021

[brb3448-bib-0090] Zhao, T. , F. Duan , X. Liao , Z. Dai , M. Cao , Y. He , et al. 2015 Test‐retest reliability of white matter structural brain networks: a multiband diffusion MRI study. Front. Hum. Neurosci. 9:59. 25741265 10.3389/fnhum.2015.00059 PMC4330899

[brb3448-bib-0091] Zhou, S. K. , and R. Chellappa . 2006 From sample similarity to ensemble similarity: probabilistic distance measures in reproducing kernel Hilbert space. IEEE Trans. Pattern Anal. Mach. Intell. 28:917–929. 16724586 10.1109/TPAMI.2006.120

[brb3448-bib-0092] Zhou, L. , Y. Wang , Y. Li , P. T. Yap , and D. Shen , Alzheimer's Disease Neuroimaging Initiative (ADNI) 2011 Hierarchical anatomical brain networks for MCI prediction: revisiting volumetric measures. PLoS One 6:e21935. 21818280 10.1371/journal.pone.0021935 PMC3139571

[brb3448-bib-0093] Zhu, W. L. , W. Wen , Y. He , A. H. Xia , K. J. Anstey , and P. Sachdev . 2012 Changing topological patterns in normal aging using large‐scale structural networks. Neurobiol. Aging 33:899–913. 20724031 10.1016/j.neurobiolaging.2010.06.022

[brb3448-bib-0094] Zielinski, B. A. , E. D. Gennatas , J. Zhou , and W. W. Seeley . 2010 Network‐level structural covariance in the developing brain. Proc. Natl Acad. Sci. USA 107:18191–18196. 20921389 10.1073/pnas.1003109107 PMC2964249

