# Supplementary material for: Single‐subject morphological brain networks: connectivity mapping, topological characterization and test–retest reliability
Source: Brain Behav. 2016 Mar 3;6(4):e00448. doi: 10.1002/brb3.448 (PMC4782249; doi:10.1002/brb3.448)
Supplement: Supplementary file 1 — Table S1. Regions of interest from the AAL atlas. Table S2. Regions of interest from the HOA atlas. [file BRB3-6-e00448-s001.docx]

**Table S1**. Regions of interest from the AAL atlas

| **Index** | **Regions** | **Abbreviations** | **Index** | **Regions** | **Abbreviations** |
| --- | --- | --- | --- | --- | --- |
| 1,2 | Superior frontal gyrus, dorsolateral | SFGdor | 47,48 | Middle frontal gyrus, orbital part | ORBmid |
| 3,4 | Middle frontal gyrus | MFG | 49,50 | Inferior frontal gyrus, orbital part | ORBinf |
| 5,6 | Inferior frontal gyrus, opercular part | IFGoperc | 51,52 | Superior frontal gyrus, medial orbital | ORBsupmed |
| 7,8 | Inferior frontal gyrus, triangular part | IFGtriang | 53,54 | Gyrus rectus | REC |
| 9, 10 | Rolandic operculum | ROL | 55,56 | Insula | INS |
| 11,12 | Supplementary motor area | SMA | 57,58 | Anterior cingulate and paracingulate gyri | ACG |
| 13,14 | Superior frontal gyrus, medial | SFGmed | 59,60 | Median cingulate and paracingulate gyri | DCG |
| 15,16 | Cuneus | CUN | 61,62 | Posterior cingulate gyrus | PCG |
| 17,18 | Lingual gyrus | LING | 63,64 | Parahippocampal gyrus | PHG |
| 19,20 | Superior occipital gyrus | SOG | 65,66 | Temporal pole: superior temporal gyrus | TPOsup |
| 21,22 | Middle occipital gyrus | MOG | 67,68 | Temporal pole: middle temporal gyrus | TPOmid |
| 23,24 | Inferior occipital gyrus | IOG | 69,70 | Olfactory cortex | OLF |
| 25,26 | Fusiform gyrus | FFG | 71,72 | Hippocampus | HIP |
| 27,28 | Superior parietal gyrus | SPG | 73,74 | Amygdala | AMYG |
| 29,30 | Inferior parietal, but supramarginal  and angular gyri | IPL | 75,76 | Caudate nucleus | CAU |
| 31,32 | Supramarginal gyrus | SMG | 77,78 | Lenticular nucleus, putamen | PUT |
| 33,34 | Angular gyrus | ANG | 79,80 | Lenticular nucleus, pallidum | PAL |
| 35,36 | Precuneus | PCUN | 81,82 | Thalamus | THA |
| 37,38 | Paracentral lobule | PCL | 83,84 | Precental gyrus | PreCG |
| 39,40 | Superior temporal gyrus | STG | 85,86 | Calcarine fissure and surrounding cortex | CAL |
| 41,42 | Middle temporal gyrus | MTG | 87,88 | Postcentral gyrus | PoCG |
| 43,44 | Inferior temporal gyrus | ITG | 89,90 | Heschl gyrus | HES |
| 45,46 | Superior frontal gyrus, orbital part | ORBsup |  |  |  |

The regions are listed in terms of a prior template of Anatomical Automatic Labeling atlas (Tzourio-Mazoyer et al., 2002). Regions of left and right hemispheres are indexed by odd and even numbers, respectively.

**Table S2.** Regions of interest from the HOA atlas

| **Index** | **Regions** | **Abbreviations** | | **Index** | **Regions** | **Abbreviations** | |
| --- | --- | --- | --- | --- | --- | --- | --- |
| 1,2 | Frontal pole | | FP | 57,58 | Cingulate gyrus, anterior division | | CGa |
| 3,4 | Insular cortex | | INS | 59,60 | Cingulate gyrus, posterior division | | CGp |
| 5,6 | Superior frontal gyrus | | F1 | 61,62 | Precuneus cortex | | PCN |
| 7,8 | Middle frontal gyrus | | F2 | 63,64 | Cuneal cortex | | CN |
| 9, 10 | Inferior frontal gyrus, pars triangularis | | F3t | 65,66 | Frontal orbital cortex | | FOC |
| 11,12 | Inferior frontal gyrus, pars opercularis | | F3o | 67,68 | Parahippocampal gyrus, anterior division | | PHa |
| 13,14 | Precentral gyrus | | PRG | 69,70 | Parahippocampal gyrus, posterior division | | PHp |
| 15,16 | Temporal pole | | TP | 71,72 | Lingual gyrus | | LG |
| 17,18 | Superior temporal gyrus, anterior division | | T1a | 73,74 | Temporal fusiform cortex, anterior division | | TFa |
| 19,20 | Superior temporal gyrus, posterior division | | T1p | 75,76 | Temporal fusiform cortex, posterior division | | TFp |
| 21,22 | Middle temporal gyrus, anterior division | | T2a | 77,78 | Temporal occipital fusiform cortex | | TOF |
| 23,24 | Middle temporal gyrus, posterior division | | T2p | 79,80 | Occipital fusiform gyrus | | OF |
| 25,26 | Middle temporal gyrus, temporooccipital part | | TO2 | 81,82 | Frontal operculum cortex | | FO |
| 27,28 | Inferior temporal gyrus, anterior division | | T3a | 83,84 | Central opercular cortex | | CO |
| 29,30 | Inferior temporal gyrus, posterior division | | T3p | 85,86 | Parietal operculum cortex | | PO |
| 31,32 | Inferior temporal gyrus, temporooccipital part | | TO3 | 87,88 | Planum polare | | PP |
| 33,34 | Postcentral gyrus | | POG | 89,90 | Heschl's gyrus (includes H1 and H2) | | H |
| 35,36 | Superior parietal lobule | | SPL | 91,92 | Planum temporale | | PT |
| 37,38 | Supramarginal gyrus, anterior division | | SGa | 93,94 | Supracalcarine cortex | | SCLC |
| 39,40 | Supramarginal gyrus, posterior division | | SGp | 95,96 | Occipital pole | | OP |
| 41,42 | Angular gyrus | | AG | 97,98 | Brain-stem | | Bst |
| 43,44 | Lateral occipital cortex, superior division | | OLs | 99,100 | Thalamus | | Thal |
| 45,46 | Lateral occipital cortex, inferior division | | OLi | 101,102 | Caudate | | Caud |
| 47,48 | Intracalcarine cortex | | CALC | 103,104 | Putamen | | Put |
| 49,50 | Frontal Medial cortex | | FMC | 105,106 | Pallidum | | Pall |
| 51,52 | Juxtapositional lobule cortex | | SMC | 107,108 | Hippocampus | | Hip |
| 53,54 | Subcallosal cortex | | SC | 109,110 | Amygdala | | Amy |
| 55,56 | Paracingulate gyrus | | PAC | 111,112 | Accumbens | | Accbns |

The regions are listed in terms of a prior probability template of Harvard-Oxford atlas (Kennedy et al., 1998; Makris et al., 1999). In the current study, a threshold of 0.25 was used to determine brain area outline. Regions of left and right hemisphere are indexed by odd and even numbers, respectively.
